# Morphological Stasis and Proteome Innovation in Cephalochordates

**DOI:** 10.3390/genes9070353

**Published:** 2018-07-16

**Authors:** László Bányai, Krisztina Kerekes, Mária Trexler, László Patthy

**Affiliations:** Institute of Enzymology, Research Centre for Natural Sciences, Hungarian Academy of Sciences, H-1117 Budapest, Hungary; banyai.laszlo@ttk.mta.hu (L.B.); kerekes.krisztina@ttk.mta.hu (K.K.); trexler.maria@ttk.mta.hu (M.T.)

**Keywords:** amphioxus, *Asymmetron*, *Branchiostoma*, domain architecture, gene prediction, genome annotation, lancelet, proteome, stasis, transcriptome

## Abstract

Lancelets, extant representatives of basal chordates, are prototypic examples of evolutionary stasis; they preserved a morphology and body-plan most similar to the fossil chordates from the early Cambrian. Such a low level of morphological evolution is in harmony with a low rate of amino acid substitution; cephalochordate proteins were shown to evolve slower than those of the slowest evolving vertebrate, the elephant shark. Surprisingly, a study comparing the predicted proteomes of Chinese amphioxus, *Branchiostoma belcheri* and the Florida amphioxus, *Branchiostoma floridae* has led to the conclusion that the rate of creation of novel domain combinations is orders of magnitude greater in lancelets than in any other Metazoa, a finding that contradicts the notion that high rates of protein innovation are usually associated with major evolutionary innovations. Our earlier studies on a representative sample of proteins have provided evidence suggesting that the differences in the domain architectures of predicted proteins of these two lancelet species reflect annotation errors, rather than true innovations. In the present work, we have extended these studies to include a larger sample of genes and two additional lancelet species, *Asymmetron lucayanum* and *Branchiostoma lanceolatum.* These analyses have confirmed that the domain architecture differences of orthologous proteins of the four lancelet species are because of errors of gene prediction, the error rate in the given species being inversely related to the quality of the transcriptome dataset that was used to aid gene prediction.

## 1. Introduction

Cephalochordates (lancelets), the basal group of extant chordates, diverged from other chordates (urochordates and vertebrates) about 550 Mya [1,2]. Lancelets are frequently referred to as living fossils because they have preserved a morphology and body-plan most similar to the fossil chordates from the early Cambrian and middle Cambrian period [3,4,5,6].

Lancelets, consisting of three genera (*Branchiostoma*, *Epigonichthys* and *Asymmetron*), are widely distributed in tropical and temperate shallow seas inhabiting inshore waters protected from wave action. Phylogenetic analyses based on amino acid sequences of protein-coding genes of amphioxus species have revealed that the *Asymmetron* clade diverged first from the common ancestor of extant lancelets, followed by the *Epigonichthys* and *Branchiostoma* clades [7]. The divergence time estimates of the various amphioxus species were shown to fit well with the time estimates for the closure of seaways by continental drift, providing a plausible explanation for the differences in the geographical distribution of the diverging species. These explanations implicitly assume that the common ancestor of the extant lancelets also existed in an epibenthic state, inhabiting sandy seafloors of inshore waters [7].

The striking conservation of the morphology in cephalochordates may be illustrated by the fact that although the Floridian *Branchiostoma floridae* and the Mediterranean *Branchiostoma lanceolatum* diverged ~100 Mya, they are very similar. The most plausible explanation for such morphological conservation is that their developmental program is unchanged. In harmony with this expectation, Somorjai and coworkers have shown that the expression patterns of key developmental genes are practically identical in *B. lanceolatum* and *B. floridae* [8]. 

The recent observation of Holland et al. [9] that eggs of *B. floridae*, when fertilized with sperm of *Asymmetron lucayanum* (or vice versa), develop through embryonic and larval stages, has provided the most extreme example of hybridization that has ever been demonstrated among multicellular animals as it occurs between species that diverged ~120–160 million years ago. Wilson and coworkers were the first to point out that the chief molecular barriers to interspecific hybridization are the regulatory system differences between the maternal and paternal genomes, which must function in concert if an interspecific zygote is to develop [10,11]. As a corollary, it was suggested that higher rates of regulatory evolution create a greater probability of developmental incompatibility between related animals [10,11]. Birds were shown to lose their potential for interspecific hybridization much slower than mammals because the rate of regulatory evolution is significantly higher in mammals than in birds [10,11,12]. Accordingly, the finding that *B. floridae* and *A. lucayanum* can give rise to interspecific hybrids also supports the notion that the rate of regulatory evolution is extremely low in the cephalochordate lineage. 

Cephalochordates are thus prototypic examples of phenotypic stasis; practically no morphological change over very long evolutionary periods. The underlying process causing phenotypic stasis is that populations well adapted to their local environment are subject to stabilizing selection as long as the local environment remains essentially unchanged, that is, the populations occupy relatively stable niches [13,14,15]. For the phenotypic stasis of lancelets, this explanation would imply that their common ancestor was well adapted to seafloors of inshore waters and that such environments (albeit on different continents) were relatively stable in the last ~100–160 million years.

Studies on the proteomes of lancelets have provided evidence compatible with the notion that they are subject primarily to stabilizing selection; analyses of the sequences of protein-coding genes of *B. floridae* and *A. lucayanum* have shown that strong purifying selection holds for the majority of genes and that these proteins are evolving more slowly than in the case of the slowest-evolving vertebrates [16,17]. 

Surprisingly, a study comparing the proteomes of the Chinese lancelet, *B. belcheri*, and the Florida lancelet, *B. floridae*, has concluded that the rate of creation of novel protein domain combinations is orders of magnitude greater in lancelets than in any other Metazoa [18]. In this study, the authors have compared the presence–absence status of protein domain combinations in proteins of various species and concluded that lancelets acquired three-fold more domain combinations than any vertebrate. According to their estimates, lancelets gained new domain pairs at a rate of >10 per Myr, a rate 10–100-fold higher than that observed in other metazoan lineages. The authors have assumed that such an unusually high rate of creation of novel protein domain combinations may be a result of continued activity of transposable elements in the lancelet lineage [18]. 

Interestingly, the unusually high rate of domain architecture differences observed when the proteomes of *B. belcheri* and *B. floridae* were compared is not accompanied by a high amino acid substitution rate. In agreement with the data of Delsuc et al. [2] and Putnam et al. [16], the authors have also found that the rate of amino-acid substitutions is much lower than in urochordates or vertebrates. Thus, whereas the low rate of amino acid substitution appears to be in harmony with phenotypic stasis and stabilizing selection in lancelets, the unusually high rate of creation of novel proteins (with novel domain combinations) is surprising as it would contradict not only the dominance of stabilizing selection, but also the general observation that high rates of creation of novel proteins are usually associated with major evolutionary innovations, such as those that accompanied the appearance of Metazoa and vertebrates [19,20].

In an earlier work, we have suggested a possible explanation for these contradictions: the differences in domain architecture of orthologous proteins of *B. belcheri* and *B. floridae* might reflect gene prediction errors rather than true innovations [21]. The plausibility of this assumption is supported by the fact that genome-annotation of intron-rich genomes is still difficult; the exact genomic structure of protein-coding genes of higher eukaryotes is correctly predicted for only about 60% of the genes [22]. Furthermore, as we have pointed out earlier, when predicted proteomes are compared, the contribution of gene prediction errors to domain architecture differences of orthologs may be far greater than those due to true gene rearrangements [23].

In the present work, we have extended our studies to include the genomes and transcriptomes of two additional lancelet species, *Asymmetron lucayanum* [17,24] and *B. lanceolatum* [25]. Furthermore, in addition to the sample of one hundred randomly selected genes analyzed in our earlier work [21], we have also performed in-depth analysis of key developmental genes that are critical for determining anteroposterior and dorvosventral patterning and left–right asymmetric development of amphioxus. The results of our analyses indicate that the domain architecture differences of orthologous proteins of the four lancelet species are due to errors of gene prediction, and that the rate of gene prediction error is inversely related to the quality of the transcriptome dataset used to aid gene prediction. The proportion of correctly predicted proteins was found to increase in the order *B. lanceolatum << B. floridae < B. belcheri < A. lucayanum*, reflecting differences in the information-content of their transcriptome datasets.

## 2. Materials and Methods 

We have randomly selected one hundred proteins of *B. belcheri* from the dataset Branchiostoma.belcheri HapV2_proteins.fa deposited on the website of the genome data of Chinese lancelet [26] as described previously [21]. 

In the present work, we have also selected key developmental genes that are known to be critical for determining anteroposterior (AP) and dorvosventral (DV) patterning, and left-right (LR) asymmetric development of amphioxus [8,27,28,29,30]. Sequences of the latter group of *B. belcheri* proteins were downloaded from the Chinese Lancelet Genome website [26]. The genes selected represent constituents of several signaling pathways (the Wnt/β-catenin, Nodal, and Bmp pathways) that pattern the dorsoventral axis; constituents of the Nodal and Bmp pathways that regulate development of left-right asymmetry; and *HOX*, *OTX*, *KROX*, and *PBX* genes that are crucial for determining anteroposterior patterning. 

We have used the reciprocal best-hit method to identify orthologs of the selected proteins, using the Branchiostoma.belcheri HapV2_proteins.fa dataset for *B. belcheri,* the National Center for Biotechnology Information’s (NCBI) non-redundant database of *B. floridae* proteins, the transcriptomes of *A. lucayanum* and *B. lanceolatum*, as well as the human section of the high quality, manually curated UniProtKB/Swiss-Prot database. As the mean identity of orthologous proteins of the Chinese and Florida lancelets was calculated to be 81.2% [18], we have used a cut off-value of 60% amino acid sequence identity as the minimum requirement for lancelet orthologs.

The domain architectures of proteins (defined as the linear sequence of Pfam-A domains) were determined using Pfam [31]. Multiple sequence alignments were obtained using Clustal Omega [32].

In cases where orthologous proteins of *B. belcheri*, *B. floridae*, *B. lanceolatum*, and *A. lucayanum* differed in domain architecture, we assumed that domain architecture deviation is more likely to reflect errors in gene prediction than true changes in gene structure [23]. To distinguish the correct and erroneous members of the ortholog pair, we have compared their domain architectures with those of their orthologs present in the UniProtKB/Swiss-Prot database; the proteins with the same domain architecture as those of orthologous Swiss-Prot entries were judged to be correctly predicted. In the case of some lancelet proteins where human orthologs could not be identified unambiguously in the Swiss-Prot database, the proteins were analyzed with the MisPred tools [33,34] to check whether they are erroneous (incomplete or mispredicted).

To decide whether the domain architecture deviation of the erroneous member(s) of the ortholog groups reflects error in gene prediction or true change in gene structure, we have re-annotated these genes using the FixPred protocol [35]. The FixPred pipeline attempts to correct mispredicted sequences by finding experimental evidence for the correct sequence version in complementary DNA (cDNA), expressed sequence tag (EST), and transcriptome databases and by re-analyses of genomic sequences.

## 3. Results

### 3.1. Absence of Orthologs in Some Lancelet Species May Reflect Errors of Gene Prediction

Out of the randomly selected proteins of *B. belcheri*, 76% had orthologs in the human section of the UniProtKB/Swiss-Prot database (Supplementary Table S1). In this dataset, 93% of the *B. belcheri* proteins had orthologs in *A. lucayanum*, 92% in *B. floridae*, but only 79% had matches in *B. lanceolatum*. If a *B. belcheri* protein had no ortholog in another lancelet species (although orthologs are present in the Swiss-Prot database), we assumed that this reflects an error of genome annotation rather than true loss of the gene. In such cases, we subjected genomic and/or transcriptomic databases to analysis by FixPred tools to find evidence for the presence of the ‘missing’ gene. Here, we illustrate this type of analysis with Ryk receptor tyrosine kinase.

#### Ryk Receptor Tyrosine Kinase

**Figure 1:**
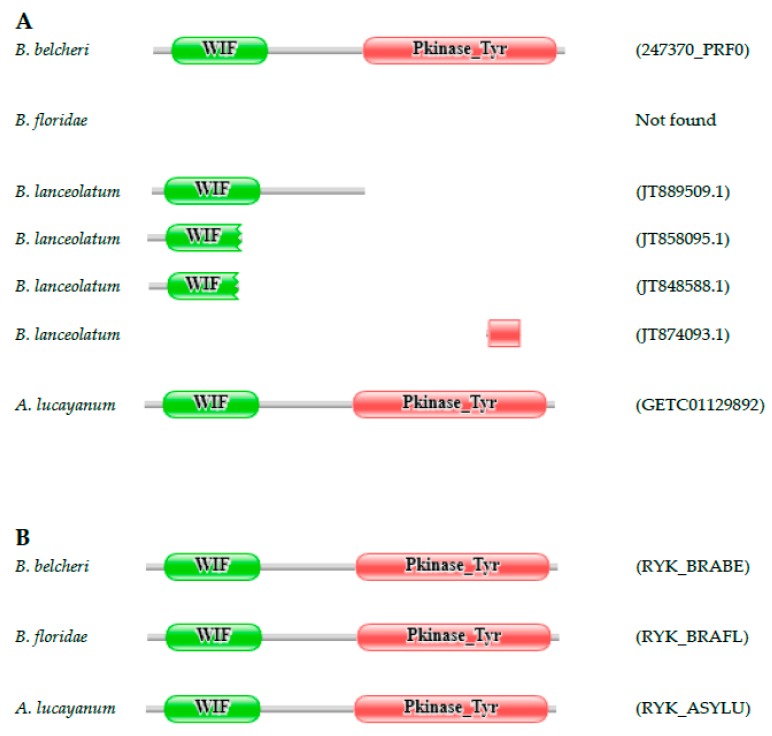
Ryk receptor tyrosine kinase genes are present in lancelet species. (**A**) Although the *Branchiostoma belcheri* genome encodes a full-length Ryk receptor tyrosine kinase (247370_PRF0), no ortholog is found in the predicted proteome of *Branchiostoma floridae*. The transcriptome of *Asymmetron lucayanum* contains transcripts encoding full-length Ryk receptor tyrosine kinase (GETC01129892.1), but in the case of *B. lanceolatum*, only fragments of the *RYK* gene are represented in its transcriptome; (**B**) Analysis of the *B. floridae* genome and transcriptome with FixPred provided evidence for a *RYK* gene that encodes a protein (RYK_BRAFL) with the same domain architecture as those of *B. belcheri* (RYK_BRABE) and *A. lucayanum* (RYK_ASYLU). The figure illustrates the Pfam domain architectures of the proteins and the position of protein fragments of the Ryk receptor tyrosine kinases. Color code for Pfam-A domains: Wnt-inhinitory factor (WIF)—green; Pkinase_Tyr—red.

Protein 247370_PRF0 of *B. belcheri* has a domain organization typical of Ryk receptor tyrosine kinases; they have a Wnt-inhinitory factor (WIF) domain that serves to bind Wnts [36]. Ryk proteins are present in all groups of Bilateria, as well as in Cnidaria [37], therefore, our observation that Ryk proteins are missing from the predicted proteome of *B. floridae* was surprising (Supplementary Table S1). Our earlier analysis of the *B. floridae* genome and transcriptome, however, provided evidence for a *RYK* gene that encodes a protein with the same domain organization as that of *B. belcheri* [21]. Analysis of the transcriptome of *A. lucayanum* identified full-length transcripts that confirm the existence and activity of a *RYK* gene in this lancelet species. The transcriptome of *B. lanceolatum* also contained transcript fragments derived from its *RYK* gene; although the full-length protein could not be reconstructed from these fragments, they confirm the existence of an active *RYK* gene in *B. lanceolatum* (Figure 1). 

### 3.2. Domain Architecture Differences of Orthologs of Lancelet Species May Reflect Errors of Gene Prediction

As we have outlined in the Materials and Methods, the rationale of our approach was the assumption that lancelet proteins with the same domain organization as those of orthologous Swiss-Prot entries are correctly predicted. In the dataset of randomly selected proteins that have orthologs in the human Swiss-Prot database (Supplementary Table S1), 69%, 58%, 51%, and 7% of the proteins of *A. lucayanum*, *B. belcheri*, *B. floridae*, and *B. lanceolatum*, respectively, had the same domain architecture as their human ortholog. 

The extremely low proportion of *B. lanceolatum* proteins with the correct domain architecture probably reflects the fact that its transcriptome represents a relatively low coverage of its gene set. The cDNA library of *B. lanceolatum*, constructed from around 1.4 million reads, could be assembled into contigs with an average length of ~490 bp [25], and such short contigs are likely to encode only fragments of proteins [25]. 

Conversely, our observation that *A. lucayanum* has the highest proportion of the proteins with the correct domain architecture is explained by the fact that its transcriptome [17] is a rich source of experimental evidence for genes expressed in various tissues of lancelets. The authors have obtained ~146 million and ~177 million reads for the adult and 20h larvae libraries and assembled them into contigs with weighted median lengths of 1886 bp and 1635 bp, respectively, thus the transcriptome of *A. lucayanum* is more likely to contain full-length transcripts of the proteins. 

Because a gene mispredicted in one of the lancelet species may be correctly predicted in another species, the percentage of lancelet proteins with correct domain architecture is higher than in any of the individual species; 76% of the ortholog groups contained at least one amphioxus protein with the correct domain architecture. 

In the dataset of proteins involved in developmental regulation (Supplementary Table S2), all the lancelet proteins had orthologs in the human UniProtKB/Swiss-Prot dataset and 92% of the ortholog groups contained at least one amphioxus protein with the correct domain architecture; 92%, 82%, and 91% of the proteins of *A. lucayanum*, *B. belcheri*, and *B. floridae*, respectively, had the same domain architecture as their human ortholog, but only a lower proportion (69%) of the orthologs had full-length transcripts in the *B. lanceolatum* transcriptome. 

We have shown that deviations in the domain organization of proteins of *B. belcheri*, *B. floridae*, or *A. lucayanum* from those of their human orthologs reflect errors of gene prediction; in these cases, FixPred found genomic and/or transcriptomic evidence for the conservation of the domain architectures. In the case of *B. lanceolatum*, however, full-length versions of the protein fragments could not be reconstructed. In summary, our data suggest that the percentage of correct primary protein predictions tends to increase in the order *B. lanceolatum* << *B. floridae* < *B. belcheri* < *A. lucayanum*, and that re-annotation with FixPred can verify the conservation of domain architectures, provided that genomic sequences are available. We can illustrate these conclusions with examples selected from Supplementary Tables S1 and S2.

#### 3.2.1. WAP, Follistatin, Immunoglobulin, Kunitz and NTR Domain-Containing (WFIKKN) Protein

**Figure 2:**
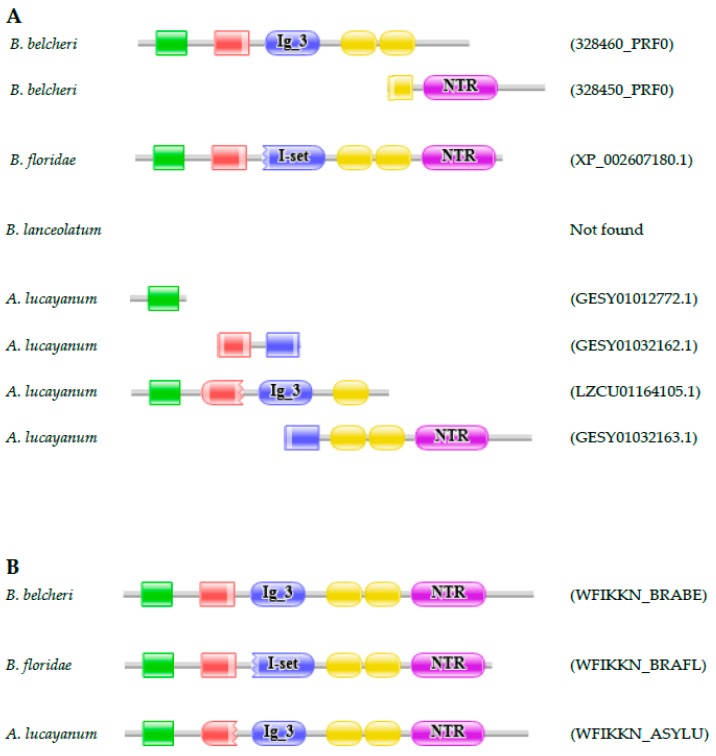
WAP, Follistatin, Immunoglobulin, Kunitz and NTR Domain-Containing (*WFIKKN*) genes encoding proteins with domain architectures typical of vertebrate WFIKKN proteins are present in the genomes of *B. belcheri*, *B. floridae* and *A. lucayanum*. (**A**) Proteins 328450_PRF0 and 328460_PRF0 of *B. belcheri* correspond to different regions of the WFIKKN protein of *B. belcheri* and the WFIKKN protein of *B. floridae* (XP_002607185.1). The transcriptome of *A. lucayanum* contains three transcripts (GESY01012772.1, GESY01032162.1, and GESY01032163.1) of the *WFIKKN* gene and the genome assembly of *A. lucayanum* contains a single scaffold encoding the 5′ part half of the *WFIKKN* gene (LZCU01164105.1); (**B**) Analysis of the transcriptome and genome of *A. lucayanum* with FixPred has provided evidence that its *WFIKKN* gene encodes a protein (WFIKKN_ASYLU) with the same domain architecture as those of *B. belcheri* (WFIKKN_BRABE) and *B. floridae* (WFIKKN_BRAFL). The figure illustrates the Pfam domain organizations of the proteins and the position of protein fragments of WFIKKN proteins. Color code for Pfam-A domains: WAP—green; Kazal—red; Immunoglobulin—blue; Kunitz—yellow; netrin (NTR)—purple.

Proteins 328450_PRF0 and 328460 PRF0 of *B. belcheri* are orthologous with the C-terminal and N-terminal parts of XP_002607180.1 of *B. floridae*, respectively [21]. Comparison of the domain structures of 328450_PRF0, 328460_PRF0, and XP_002607180.1 with those of their human orthologs has revealed that XP_002607180.1 of *B. floridae* has the domain organization typical of WFIKKN proteins of vertebrates (Figure 2). The ‘fragmentation’ of the WFIKKN protein of *B. belcheri* was shown to be the result of an error of the prediction of the *B. belcheri* proteome that could be corrected by re-annotation of the *B. belcheri WFIKKN* gene [21]. The transcriptome of *B. lanceolatum* had no transcript derived from its *WFIKKN* gene. On the other hand, the transcriptome of *A. lucayanum* contains three transcripts (GESY01032163.1, GESY01032162.1, and GESY01012772.1) of the *WFIKKN* gene and the genome assembly contained a single scaffold containing the 5′ part half of the *WFIKKN* gene (Sequence ID: LZCU01164105.1), permitting the reconstruction of the full-length sequence using the FixPred protocol. Analysis of the genomes and transcriptomes of *B. belcheri*, *B. floridae*, and *A. lucayanum* thus confirm that the domain architectures of WFIKKN proteins are conserved in lancelets.

#### 3.2.2. Calcium-Binding Mitochondrial Carrier Protein 

**Figure 3:**
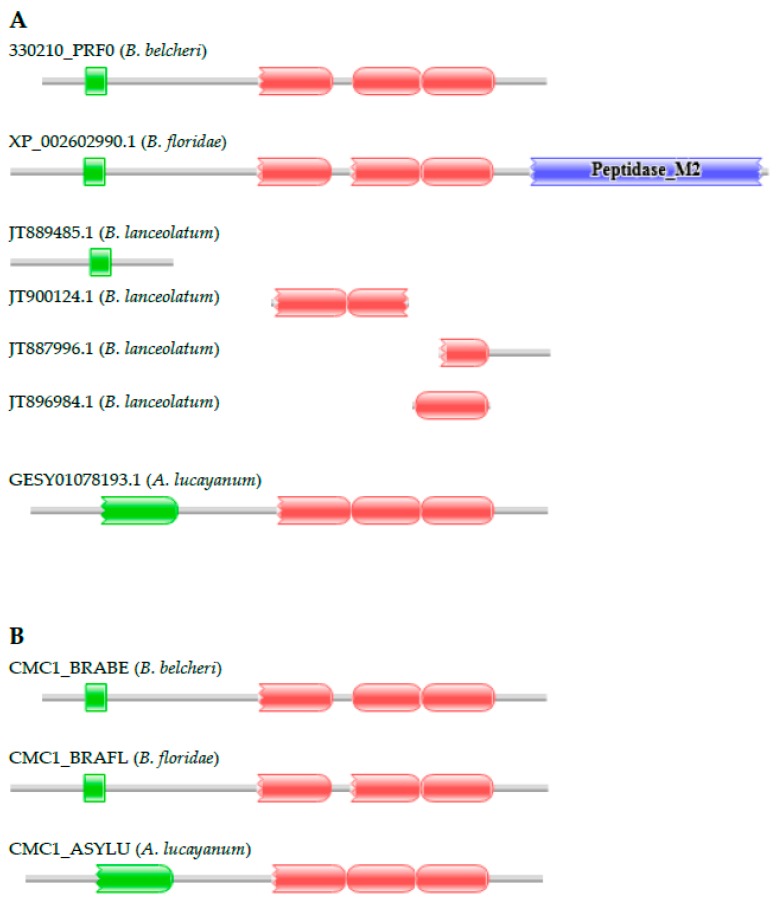
Genes encoding proteins with domain architectures typical of vertebrate calcium-binding mitochondrial carrier proteins are present in the genomes of *B. belcheri*, *B. floridae*, *B. lanceolatum*, and *A. lucayanum*. (**A**) The *B. floridae* protein XP_002602990.1 is an ortholog of the calcium-binding mitochondrial carrier protein of *B. belcheri* (330210_PRF0), but it contains a C-terminal extension with a Peptidase_M2 domain. The transcriptome of *A. lucayanum* contains full-length transcripts of the gene (GESY01078193.1) encoding a protein with a domain architecture characteristic of calcium-binding mitochondrial carrier proteins. The transcriptome of *B. lanceolatum* contains several transcripts of the calcium-binding mitochondrial carrier protein gene and one of them, matching the C-terminal part of the protein (JT887996.1), supports the conclusion that it does not have a C-terminal Peptidase_M2 domain; (**B**) The corrected sequence (CMC1_BRAFL) of the calcium-binding mitochondrial carrier protein of *B. floridae* [21] has the same domain architecture as its orthologs of *B. belcheri* (CMC1_BRABE) and *A. lucayanum* (CMC1_ASYLU). The figure illustrates the Pfam domain architectures of the proteins and the position of protein fragments of calcium-binding mitochondrial carrier proteins. Color code for Pfam-A domains: EF-hand—green; Mito_carr—red; Peptidase_M2—blue.

The 330210_PRF0 protein of *B. belcheri* has the domain architecture typical of calcium-binding mitochondrial carrier proteins, containing an EF-hand domain and three Mito_carr domains (Supplementary Table S1). The predicted *B. floridae* orthologue (XP_002602990.1) differs in domain architecture in that it has a C-terminal extension with a Peptidase_M2 domain. Because the XP_002602990.1 protein sequence violated basic dogmas that MisPred uses to detect gene prediction errors [33,34], we suspected that the *B. floridae* protein is mispredicted. Analyses of EST databases have confirmed this suspicion; ESTs originating from the putative ‘fusion’ region were found to align either with the N-terminal or the C-terminal fusion parts, but not both parts of the chimeric XP_002602990.1 protein, permitting the correction of the sequence [21]. Our conclusion that lancelets contain classical calcium-binding mitochondrial carrier proteins is also supported by the analysis of the transcriptome of *A. lucayanum*; it contains full-length transcripts (e.g., GETC01125846.1) of the calcium-binding mitochondrial carrier protein gene (Figure 3). The transcriptome of *B. lanceolatum* had several transcript fragments derived from its calcium-binding mitochondrial carrier protein gene. Although these fragments did not permit the reconstruction of the full-length protein, one of them matching the C-terminal part of the protein (JT887996.1) supports the conclusion that the protein does not contain a Peptidase_M2 domain as it has a stop codon in the same region as its orthologs in the other lancelet species. Analysis of the genomes and transcriptomes of lancelets thus confirms that the domain organizations of calcium-binding mitochondrial carrier proteins are conserved in lancelets.

#### 3.2.3. Histone H2A Deubiquitinase 

**Figure 4:**
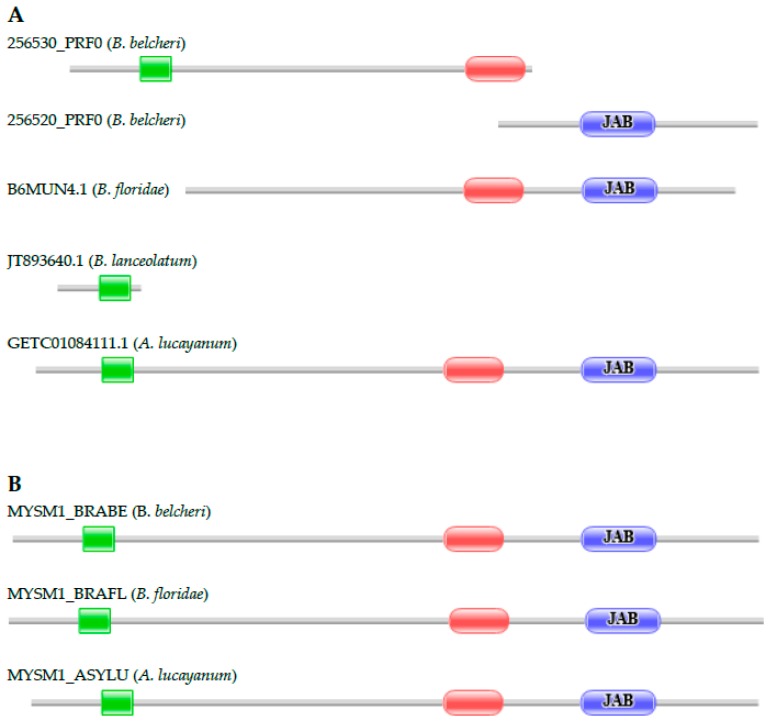
Myb Like, SWIRM And MPN Domains 1 (MYSM1) genes encoding proteins with domain architectures typical of histone H2A deubiquitinases are present in the genomes of *B. belcheri*, *B. floridae*, and *A. lucayanum*. (**A**) The architectures of the predicted histone H2A deubiquitinases of *B. belcheri* and *B. floridae* deviate from the conserved domain architecture of these proteins in that one of the fragments of the *B. belcheri* protein (256530_PRF0) lacks the JAB domain, another fragment of the *B. belcheri* ortholog (256520_PRF0) lacks the Myb_DNA-binding and SWIRM domains, whereas the *B. floridae* protein (B6MUN4.1) lacks the Myb_DNA-binding domain. The transcriptome of *A. lucayanum* contains full-length transcripts of the MYSM1 gene, but the transcriptome of *B. lanceolatum* had only one transcript fragment derived from its MYSM1 gene; (**B**) Re-annotation of the *MYSM1* genes of *B. belcheri* and *B. floridae* with the FixPred protocol has shown that these deviations are due errors in gene prediction, permitting the correction of these sequences (MYSM1_BRABE, MYSM1_BRAFL). The figure illustrates the Pfam domain architectures of the proteins and position of protein fragments of MYSM1 proteins. Color code for Pfam-A domains: Myb_DNA-binding—green; SWIRM—red; JAB—blue.

Histone H2A deubiquitinases are conserved proteins involved in the regulation of nucleosome dissociation and transcription activation. The domain architecture of the human protein Q5VVJ2 (MYSM1_HUMAN) consists of a Myb_DNA-binding, a SWIRM, and a JAB domain. The architectures of the predicted *B. belcheri* and *B. floridae* proteins, however, differed from that of the human ortholog in as much as one of the *B. belcheri* orthologs fragments (256530_PRF0) lacks the JAB domain, another ortholog fragment (256520_PRF0) lacks the Myb_DNA-binding and SWIRM domains, whereas the *B. floridae* protein (B6MUN4.1) lacks the Myb_DNA-binding domain. Our analyses have confirmed that these deviations in domain architecture reflect errors in gene prediction that could be corrected with FixPred (Figure 4). The conclusion that histone H2A deubiquitinases of lancelets retained the domain architecture characteristic of Myb Like, SWIRM And MPN Domains 1 (*MYSM1*) genes is also supported by the analysis of the transcriptome of *A. lucayanum*; it contains full-length transcripts (e.g., GETC01084111.1) of the *MYSM1* gene. The transcriptome of *B. lanceolatum* had only one transcript fragment derived from its *MYSM1* gene. Analysis of the genomes and transcriptomes of *B. belcheri*, *B. floridae*, and *A. lucayanum* thus confirm that the domain architectures of MYSM1 proteins are conserved in lancelets.

#### 3.2.4. Chordin

**Figure 5:**
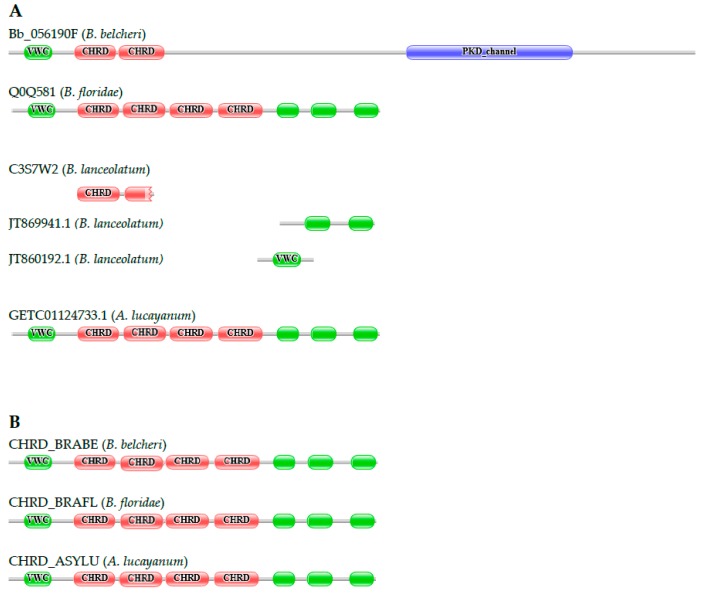
Chordin (*CHRD*) genes encoding proteins with domain architectures typical of vertebrate chordins are present in the genomes of *B. belcheri*, *B. floridae*, and *A. lucayanum*. (**A**) The characteristic domain architecture of vertebrate chordins is conserved in *B. floridae* (Q0Q581), but the structure of the *B. belcheri* ortholog (Bb_056190F) is markedly different in that it lacks some of the domains and has a long C-terminal extension containing a polycystic kidney disease (PKD)_channel domain. The transcriptome of *A. lucayanum* contains full-length transcripts of the *CHRD* gene (GETC01124733.1). The transcriptome of *B. lanceolatum* had several transcripts of the *CHRD* gene and one of them matching the C-terminal part of the protein (JT869941.1) supports the conclusion that the protein does not have a PKD_channel domain; (**B**) Re-annotation of the *B. belcheri CHRD* gene with the MisPred/FixPred protocol has shown that these deviations are due to errors in gene prediction, permitting the correction of this sequence (CHRD_BRABE). The figure illustrates the Pfam domain architectures of the proteins and position of protein fragments of chordins. Color code for Pfam-A domains: von Willebrand factor type C domain (VWC)—green; chordin (CHRD)—red; PKD_channel—blue.

Chordin is a key developmental protein that dorsalizes early chordate embryonic tissues by binding to ventralizing TGF-β family bone morphogenetic proteins and sequestering them in latent complexes. The domain architecture of the human protein is conserved in *B. floridae* (Q0Q581_BRAFL), the domain architecture of the predicted sequence of the *B. belcheri* ortholog (Bb_056190F), however, is markedly different; it lacks two of the chordin (CHRD) domains, the C-terminal von Willebrand factor type C domain (VWC) domain is missing, the penultimate VWC domain is truncated, and the protein has a long C-terminal extension containing a polycystic kidney disease (PKD)_channel domain (Figure 5). Because the protein architecture of Bb_056190F violates basic dogmas of MisPred, we have suspected that the difference between the domain organizations of the *B. belcheri* and *B. floridae* proteins reflects an error of the prediction of the *B. belcheri* protein and not true innovation. Re-annotation of the *CHRD* gene of *B. belcheri* with the FixPred protocol has shown that this deviation is due to an error in gene prediction, permitting the correction of this sequence. Our conclusion that lancelets contain ‘classical’ chordins is also supported by the analysis of the transcriptome of *A. lucayanum*; it contains full-length transcripts (e.g., GETC01124733.1) of the *CHRD* gene. The transcriptome of *B. lanceolatum* had several transcript fragments derived from its *CHRD* gene. Although these fragments did not permit the reconstruction of the full-length protein, an EST (JT869941.1) matching the C-terminal part of the protein supports the conclusion that the protein does not contain a PKD_channel domain; it has a stop codon in the same region as its orthologs in the other lancelet species. Analysis of the genomes and transcriptomes of *B. belcheri*, *B. floridae*, and *A. lucayanum* thus confirm that the domain architectures of chordins are conserved in lancelets.

### 3.3. Lancelet Proteins with Domain Architectures Apparently Unique to Cephalochordates

Twenty-four percent of the randomly selected proteins of *B. belcheri* orthologs had domain architectures with no clear equivalents in the human section of the UniProtKB/Swiss-Prot database (Supplementary Table S3). These proteins belong to two categories: they are variants of vertebrate-specific proteins (e.g., the plasminogen-related protein 040970_PFF0 and its orthologs) or they are epaktologs, proteins only related through shared domains [38] rather than orthologs of human proteins. 

There are several proteins in Supplementary Table S3 that appear to be correctly predicted as suggested by the observation that they pass the quality control of MisPred and that the orthologs from three lancelet species have identical domain architectures. 

It must be emphasized, however, that the identity of domain architectures of lancelet orthologs, per se, does not guarantee that they are valid lancelet-specific innovations. Because of the similarity of lancelet genomes and the similarity of gene-prediction protocols, genome annotation may yield similarly erroneous predictions. This danger has been illustrated with the case of protein 018300_PFF0 of *B. belcheri* and its *B. floridae* ortholog, XP_002588697.1 that contains a Lectin_C domain and five tandem fibronectin type II (fn2) domains [21]. 

## 4. Discussion

The ENCODE Genome Annotation Assessment Project [39] has shown that—although none of the gene prediction strategies produce perfect predictions—evidence-based prediction methods exploiting experimental data (protein, messenger RNA, ESTs, etc.) were the most accurate, suggesting that the reliability of gene predictions increases as a function of the information-content of the experimental datasets.

The original gene models of *B. floridae* and *B. belcheri* analyzed in the present work have been predicted by integrating the results obtained by de novo, homology-based, and transcriptome-based gene prediction methods [16,18]. In the case of *B. belcheri*, prediction was aided by ~300 million ESTs [18], whereas in the case of *B. floridae* gene, models were based on only about 480,000 ESTs [16]. Oulion and coworkers [25] have also pointed out the relatively low coverage of lancelet genomes by the *B. floridae* transcriptome; whereas 83% of the predicted genes of the *B. floridae* complete genome sequence are represented in the *B. lanceolatum* transcriptome, only 41% of the genes were found to match the much smaller dataset from the *B. floridae* transcriptome [40]. 

Accordingly, it is likely that as the *B. floridae* gene models were based on a significantly smaller dataset of ESTs than gene models of *B. belcheri*, misprediction may be more severe in the case of *B. floridae* genes. In harmony with this expectation, we have found that a higher proportion of *B. belcheri* proteins have the same domain architecture as their human Swiss-Prot orthologs than in the case *B. floridae* proteins (see Section 3.2). 

The uncertainty in gene predictions is also reflected by the fact that, whereas 30,392 protein-coding genes were predicted for the Chinese lancelet genome [18], the Florida lancelet genome was predicted to contain only 21,900 protein-coding genes [16]. It must be emphasized that both gene number estimates were based on draft genomes, thus genes are likely to be fragmented onto several contigs, which is known to cause significant errors in gene prediction [41].

In a recent study, Francis and Wörheide have also pointed out major problems with the original genome assembly and genome annotation of the *B. floridae* genome [42]. These authors have shown that, regardless of genome size, the ratio of introns to intergenic sequence is comparable across essentially all animals. Whereas correctly assembled, properly annotated genomes of model organisms have intron to intergenic sequence ratios close to 1:1, this does not hold for the genome of the lancelet *B. floridae*. The original gene models [16] had annotated almost 90% of the genome as genes, assigning the majority (85%) of the genome to introns. Through re-annotation of the *B. floridae* scaffolds using the reads from a paired-end RNA sequencing (RNAseq) library, these authors have shown that more of the genome is intergenic than intronic, suggesting that the original genome annotation has erroneously fused or missed many genes. 

The transcriptome of *A. lucayanum* [17] provided so far the richest source of data for evidence-based estimation of the number of genes in lancelet genomes. Based on the transcriptome of *A. lucayanum*, Yue et al. have identified 23,245 coding sequences (CDSs) (adult library) and 22,941 CDSs (20-h larvae library) of *A. lucayanum*, respectively [17,24]. The authors have also re-annotated the genome assembly of *B. floridae*, leading them to conclude that this lancelet genome contains 28,586 genes [17], a number significantly higher than the number (~22,000) originally predicted [16].

The results of our analyses confirm that the domain architecture differences of orthologous proteins of various lancelet species reflect errors of gene prediction; the proportion of correctly predicted proteins increases in the order *B. lanceolatum << B. floridae < B. belcheri < A. lucayanum*, reflecting differences in the information-content of their transcriptomes. 

## Supplementary Materials

The following are available online at http://www.mdpi.com/2073-4425/9/7/353/s1, Table S1: Comparison of the domain architectures of randomly selected lancelet proteins with those of their human Swiss-Prot orthologs, Table S2: Comparison of the domain architectures of key developmental proteins of lancelets with those of their human Swiss-Prot orthologs, Table S3: Comparison of the domain architectures of randomly selected lancelet proteins with novel domain architectures.
