# Supplementary material for: Morphological Stasis and Proteome Innovation in Cephalochordates
Source: Genes (Basel). 2018 Jul 16;9(7):353. doi: 10.3390/genes9070353 (PMC6071037; doi:10.3390/genes9070353)
Supplement: Supplementary file 1 [file genes-09-00353-s001.zip › genes-314079-supplementary-table S1.docx]

Supplementary Material: Morphological Stasis and Proteome Innovation
in Cephalochordates

László Bányai, Krisztina Kerekes, Mária Trexler and László Patthy

**Table S1.** Comparison of the domain architectures of randomly selected lancelet proteins with those of their human Swiss-Prot orthologs. Proteins of *Branchiostoma* *belcheri*, containing at least two Pfam-A domains, were randomly selected and their lancelet and human Swiss-Prot orthologs were identified by the reciprocal best-hit method. Domain architectures, defined as the linear sequence of Pfam-A domains, were determined with Pfam. The first five columns of the table list the sequence identities (IDs) of pedicted proteins (*B. belcheri* and *B. floridae*), sequence IDs of trancripts of orthologous protein-coding genes (*Asymmetron lucayanum* and *B. lanceolatum*), and IDs of human Swiss-Prot entries. The last five columns of the table indicate the PfamA domains present in the various entries. Cells containing entries whose domain architecture is identical with those of orthologs in the Swiss-Prot database are highlighted in green.

| **Sequence ID** | | | | | **Domain architecture** | | | | |
| --- | --- | --- | --- | --- | --- | --- | --- | --- | --- |
| ***B. belcheri*** | ***B. floridae*** | ***A. lucayanum*** | ***B. lanceolatum*** | ***H. sapiens*** | ***B. belcheri*** | ***B. floridae*** | ***A. lucayanum*** | ***B. lanceolatum*** | ***H. sapiens*** |
| 001440_PFF0 | XP_002598316.1 | GETC01094825.1 | JT883544.1 | P13674.2 | P4Ha_N | P4Ha_N | P4Ha_N |  | P4Ha_N |
|  |  |  |  |  | 2OG-FeII_Oxy_3 | 2OG-FeII_Oxy_3 | 2OG-FeII_Oxy_3 | 2OG-FeII_Oxy_3 | 2OG-FeII_Oxy_3 |
|  |  |  |  |  |  |  |  |  |  |
|  |  |  | JT857022.1 | P13674.2 |  |  |  | P4Ha_N | P4Ha_N |
|  |  |  |  |  |  |  |  |  | 2OG-FeII_Oxy_3 |
|  |  |  |  |  |  |  |  |  |  |
| 002260_PFF0 | XP_002598117.1 | GETC01117156.1 | JT882468.1 | Q13705.3 | Activin_recp | Activin_recp | Activin_recp |  | Activin_recp |
|  |  |  |  |  | Pkinase_ Tyr | Pkinase_ Tyr | Pkinase_ Tyr | Pkinase_ Tyr | Pkinase_ Tyr |
|  |  |  |  |  |  |  |  |  |  |
|  |  |  | JT882894.1 | Q13705.3 |  |  |  | Activin_recp | Activin_recp |
|  |  |  |  |  |  |  |  |  | Pkinase_ Tyr |
|  |  |  |  |  |  |  |  |  |  |
| 003500_PFF0 | XP_002598733.1 | GESY01076595.1 |  | A8K7I4.3 | CLCA | CLCA | CLCA |  | CLCA |
|  |  |  |  |  | VWA_2 | VWA_2 | VWA_2 |  | VWA_2 |
|  |  |  |  |  |  |  |  |  |  |
| 003850_PFF0 | XP_002597871.1 | GESY01078565.1 | JT895160.1 | O14672.1 | Pep_M12B_propep | Pep_M12B_propep | Pep_M12B_propep | Pep_M12B_propep | Pep_M12B_propep |
|  |  |  |  |  | Reprolysin_5 | Reprolysin_5 | Reprolysin_5 |  | Reprolysin_5 |
|  |  |  |  |  |  | Pep_M12B_propep |  |  |  |
|  |  |  |  |  | Disintegrin | Disintegrin | Disintegrin |  | Disintegrin |
|  |  |  |  |  |  |  |  |  |  |
| 012350_PFF0 | XP_002604729.1 | GETC01090945.1 | JT884420.1 | Q92563.1 | Kazal_2 | Kazal_2 | Kazal_2 |  | Kazal_2 |
|  |  |  |  |  | SPARC_Ca_bdg | SPARC_Ca_bdg | SPARC_Ca_bdg | SPARC_Ca_bdg | SPARC_Ca_bdg |
|  |  |  |  |  | Thyroglobulin_1 | Thyroglobulin_1 | Thyroglobulin_1 | Thyroglobulin_1 | Thyroglobulin_1 |
|  |  |  |  |  |  |  |  |  |  |
|  |  |  | JT884847.1 | Q92563.1 |  |  |  | Kazal_2 | Kazal_2 |
|  |  |  |  |  |  |  |  |  | SPARC_Ca_bdg |
|  |  |  |  |  |  |  |  |  | Thyroglobulin_1 |
|  |  |  |  |  |  |  |  |  |  |
| 052490_PFF0 | XP_002608045.1 | GESY01046504.1 | JT898612.1 | Q96HF1.2 | Fz | Fz | Fz |  | Fz |
|  |  |  |  |  | NTR | NTR | NTR |  | NTR |
|  |  |  |  |  |  |  |  |  |  |
| 058270_PFF0 | XP_002586055.1 | GETC01098073.1 | JT899677.1 | Q01973.2 | I-set | I-set | I-set |  | I-set |
|  |  |  |  |  | Fz | Fz | Fz |  | Fz |
|  |  |  |  |  | Kringle | Kringle | Kringle |  | Kringle |
|  |  |  |  |  |  |  | I-set |  |  |
|  |  |  |  |  |  | Fz |  |  |  |
|  |  |  |  |  |  | Kringle |  |  |  |
|  |  |  |  |  |  | I-set |  |  |  |
|  |  |  |  |  |  | Pkinase_Tyr | Pkinase_Tyr | Pkinase_Tyr | Pkinase_Tyr |
|  |  |  |  |  |  | I-set |  |  |  |
|  |  |  |  |  |  | Pkinase_Tyr |  |  |  |
|  |  |  |  |  |  |  |  |  |  |
|  |  |  | JT886650.1 | Q01973.2 |  |  |  |  | I-set |
|  |  |  |  |  |  |  |  | Fz | Fz |
|  |  |  |  |  |  |  |  |  | Kringle |
|  |  |  |  |  |  |  |  |  |  |
|  |  |  |  |  |  |  |  |  |  |
|  |  |  |  |  |  |  |  |  |  |
|  |  |  |  |  |  |  |  |  |  |
|  |  |  |  |  |  |  |  | Pkinase_Tyr | Pkinase_Tyr |
|  |  |  |  |  |  |  |  |  |  |
| 078830_PFF0 | XP_002596432.1 | GETC01136904.1 | JT866061.1 | P11498.2 | Biotin_carb_N | CPSase_L_chain | Biotin_carb_N |  | Biotin_carb_N |
|  |  |  |  |  | CPSase_L_D2 | CPSase_L_D2 | CPSase_L_D2 |  | CPSase_L_D2 |
|  |  |  |  |  | Biotin_carb_C | Biotin_carb_C | Biotin_carb_C | Biotin_carb_C | Biotin_carb_C |
|  |  |  |  |  | HMGL-like | HMGL-like | HMGL-like |  | HMGL-like |
|  |  |  |  |  | PYC_OADA | PYC_OADA | PYC_OADA |  | PYC_OADA |
|  |  |  |  |  | Biotin_lipoyl | Biotin_lipoyl | Biotin_lipoyl |  | Biotin_lipoyl |
|  |  |  |  |  |  |  |  |  |  |
|  |  |  | JT862762.1 | P11498.2 |  |  |  | Biotin_carb_N | Biotin_carb_N |
|  |  |  |  |  |  |  |  |  | CPSase_L_D2 |
|  |  |  |  |  |  |  |  |  | Biotin_carb_C |
|  |  |  |  |  |  |  |  |  | HMGL-like |
|  |  |  |  |  |  |  |  |  | PYC_OADA |
|  |  |  |  |  |  |  |  |  | Biotin_lipoyl |
|  |  |  |  |  |  |  |  |  |  |
| 080250_PFF0 | XP_002598506.1 | GETC01128999.1 |  | Q9GZT5.1 | LRR_8 |  |  |  |  |
|  |  |  |  |  | I-set |  |  |  |  |
|  |  |  |  |  | Wnt | Wnt | Wnt |  | Wnt |
|  |  |  |  |  |  |  |  |  |  |
|  | XP_002598508.1 |  |  | Q9GZT5.1 |  | Wnt |  |  | Wnt |
|  |  |  |  |  |  |  |  |  |  |
| 091850_PFF0 | XP_002591393.1 | GETC01136806.1 | JT868378.1 | O95490.2 | Gal_Lectin | Gal_Lectin | Gal_Lectin | Gal_Lectin | Gal_Lectin |
|  |  |  |  |  | OLF | OLF | OLF |  | OLF |
|  |  |  |  |  | HRM | HRM | HRM |  | HRM |
|  |  |  |  |  | GAIN | GAIN | GAIN |  | GAIN |
|  |  |  |  |  | GPS |  | GPS |  | GPS |
|  |  |  |  |  | 7tm_2 | 7tm_2 | 7tm_2 |  | 7tm_2 |
|  |  |  |  |  |  |  |  |  | Latrophilin |
|  |  |  |  |  |  |  |  |  |  |
|  |  |  | JT868759.1 | O95490.2 |  |  |  |  | Gal_Lectin |
|  |  |  |  |  |  |  |  |  | OLF |
|  |  |  |  |  |  |  |  |  | HRM |
|  |  |  |  |  |  |  |  | GAIN | GAIN |
|  |  |  |  |  |  |  |  |  | GPS |
|  |  |  |  |  |  |  |  |  | 7tm_2 |
|  |  |  |  |  |  |  |  |  | Latrophilin |
|  |  |  |  |  |  |  |  |  |  |
|  |  |  | JT890563.1 | O95490.2 |  |  |  |  | Gal_Lectin |
|  |  |  |  |  |  |  |  |  | OLF |
|  |  |  |  |  |  |  |  |  | HRM |
|  |  |  |  |  |  |  |  |  | GAIN |
|  |  |  |  |  |  |  |  |  | GPS |
|  |  |  |  |  |  |  |  | 7tm_2 | 7tm_2 |
|  |  |  |  |  |  |  |  |  | Latrophilin |
|  |  |  |  |  |  |  |  |  |  |
| 102470_PFF0 | XP_002608336.1 | GETC01132168.1 | JT881815.1 | P30876.1 | RNA_pol_Rpb2_1 | RNA_pol_Rpb2_1 | RNA_pol_Rpb2_1 | RNA_pol_Rpb2_1 | RNA_pol_Rpb2_1 |
|  |  |  |  |  | RNA_pol_Rpb2_2 | RNA_pol_Rpb2_2 | RNA_pol_Rpb2_2 | RNA_pol_Rpb2_2 | RNA_pol_Rpb2_2 |
|  |  |  |  |  | RNA_pol_Rpb2_3 | RNA_pol_Rpb2_3 | RNA_pol_Rpb2_3 | RNA_pol_Rpb2_3 | RNA_pol_Rpb2_3 |
|  |  |  |  |  | RNA_pol_Rpb2_4 | RNA_pol_Rpb2_4 | RNA_pol_Rpb2_4 |  | RNA_pol_Rpb2_4 |
|  |  |  |  |  | RNA_pol_Rpb2_5 | RNA_pol_Rpb2_5 | RNA_pol_Rpb2_5 |  | RNA_pol_Rpb2_5 |
|  |  |  |  |  | RNA_pol_Rpb2_6 | RNA_pol_Rpb2_6 | RNA_pol_Rpb2_6 |  | RNA_pol_Rpb2_6 |
|  |  |  |  |  | RNA_pol_Rpb2_7 |  | RNA_pol_Rpb2_7 |  | RNA_pol_Rpb2_7 |
|  |  |  |  |  |  |  |  |  |  |
|  |  |  | JT895824.1 | P30876.1 |  |  |  |  | RNA_pol_Rpb2_1 |
|  |  |  |  |  |  |  |  |  | RNA_pol_Rpb2_2 |
|  |  |  |  |  |  |  |  |  | RNA_pol_Rpb2_3 |
|  |  |  |  |  |  |  |  | RNA_pol_Rpb2_4 | RNA_pol_Rpb2_4 |
|  |  |  |  |  |  |  |  | RNA_pol_Rpb2_5 | RNA_pol_Rpb2_5 |
|  |  |  |  |  |  |  |  |  | RNA_pol_Rpb2_6 |
|  |  |  |  |  |  |  |  |  | RNA_pol_Rpb2_7 |
|  |  |  |  |  |  |  |  |  |  |
|  |  |  | JT903141.1 | P30876.1 |  |  |  |  | RNA_pol_Rpb2_1 |
|  |  |  |  |  |  |  |  |  | RNA_pol_Rpb2_2 |
|  |  |  |  |  |  |  |  |  | RNA_pol_Rpb2_3 |
|  |  |  |  |  |  |  |  |  | RNA_pol_Rpb2_4 |
|  |  |  |  |  |  |  |  |  | RNA_pol_Rpb2_5 |
|  |  |  |  |  |  |  |  | RNA_pol_Rpb2_6 | RNA_pol_Rpb2_6 |
|  |  |  |  |  |  |  |  |  | RNA_pol_Rpb2_7 |
|  |  |  |  |  |  |  |  |  |  |
|  |  |  | JT848270.1 | P30876.1 |  |  |  |  | RNA_pol_Rpb2_1 |
|  |  |  |  |  |  |  |  |  | RNA_pol_Rpb2_2 |
|  |  |  |  |  |  |  |  |  | RNA_pol_Rpb2_3 |
|  |  |  |  |  |  |  |  |  | RNA_pol_Rpb2_4 |
|  |  |  |  |  |  |  |  |  | RNA_pol_Rpb2_5 |
|  |  |  |  |  |  |  |  |  | RNA_pol_Rpb2_6 |
|  |  |  |  |  |  |  |  | RNA_pol_Rpb2_7 | RNA_pol_Rpb2_7 |
|  |  |  |  |  |  |  |  |  |  |
| 103400_PFF0 | XP_002593834.1 | GETC01087495.1 | JT879377.1; JT898004.1 | Q7Z5J8.3 | Ank |  | Ank |  |  |
|  |  |  |  |  | Ank | Ank | Ank |  | Ank |
|  |  |  |  |  | Arm | Arm | Arm |  | Arm |
|  |  |  |  |  | Arm | Arm | Arm |  |  |
|  |  |  |  |  |  |  |  |  |  |
|  |  | GETC01094246.1 |  | Q7Z5J8.3 |  |  |  |  |  |
|  |  |  |  |  |  |  |  |  | Ank |
|  |  |  |  |  |  |  |  |  | Arm |
|  |  |  |  |  |  |  |  |  |  |
|  |  |  |  |  |  |  |  |  |  |
| 114280_PFF0 | XP_002608942.1 | GETC01136050.1 | JT900613.1 | O75923.1 | C2 | C2 | C2 |  | C2 |
|  |  |  |  |  | C2 | C2 | C2 |  | C2 |
|  |  |  |  |  | FerI | FerI | FerI |  | FerI |
|  |  |  |  |  | C2 | C2 | C2 |  | C2 |
|  |  |  |  |  |  |  | FerA | FerA | FerA |
|  |  |  |  |  | FerB | FerB | FerB |  | FerB |
|  |  |  |  |  | C2 | C2 | C2 |  | C2 |
|  |  |  |  |  | C2 | C2 | C2 |  | C2 |
|  |  |  |  |  | C2 | C2 | C2 |  | C2 |
|  |  |  |  |  | C2 | C2 | C2 |  | C2 |
|  |  |  |  |  | Ferlin_C | Ferlin_C | Ferlin_C |  | Ferlin_C |
|  |  |  |  |  |  |  |  |  |  |
|  |  |  | JT898638.1 | O75923.1 |  |  |  |  | C2 |
|  |  |  |  |  |  |  |  |  | C2 |
|  |  |  |  |  |  |  |  |  | FerI |
|  |  |  |  |  |  |  |  |  | C2 |
|  |  |  |  |  |  |  |  |  | FerA |
|  |  |  |  |  |  |  |  |  | FerB |
|  |  |  |  |  |  |  |  |  | C2 |
|  |  |  |  |  |  |  |  |  | C2 |
|  |  |  |  |  |  |  |  | C2 | C2 |
|  |  |  |  |  |  |  |  | C2 | C2 |
|  |  |  |  |  |  |  |  |  | Ferlin_C |
|  |  |  |  |  |  |  |  |  |  |
| 117350_PFF0 | XP_002599461.1 | GESY01033161.1 |  | O14793.1/O95390.1 | TGFb_propeptide | TGFb_propeptide | TGFb_propeptide |  | TGFb_propeptide |
|  |  |  |  |  | TGF_beta | TGF_beta | TGF_beta |  | TGF_beta |
|  |  |  |  |  |  |  |  |  |  |
| 118940_PFF0 | XP_002587581.1 | GESY01082246.1 | JT878152.1 | Q6JQN1.1 | HAD_2 | HAD_2 | HAD_2 |  | HAD_2 |
|  |  |  |  |  | APH | APH | APH |  | APH |
|  |  |  |  |  | Acyl-CoA_dh_N | Acyl-CoA_dh_N | Acyl-CoA_dh_N |  | Acyl-CoA_dh_N |
|  |  |  |  |  | Acyl-CoA_dh_M | Acyl-CoA_dh_M | Acyl-CoA_dh_M |  | Acyl-CoA_dh_M |
|  |  |  |  |  | Acyl-CoA_dh_1 | Acyl-CoA_dh_1 | Acyl-CoA_dh_1 | Acyl-CoA_dh_1 | Acyl-CoA_dh_1 |
|  |  |  |  |  |  |  |  |  |  |
| 123650_PFF0 | XP_002603715.1 | GETC01131892.1 |  | Q14203.3 | CAP_GLY | CAP_GLY | CAP_GLY |  | CAP_GLY |
|  |  |  |  |  | Dynactin |  | Dynactin |  | Dynactin |
|  |  |  |  |  |  |  |  |  |  |
|  | XP_002603716.1 |  |  | Q14203.3 |  |  |  |  | CAP_GLY |
|  |  |  |  |  |  | Dynactin |  |  | Dynactin |
|  |  |  |  |  |  |  |  |  |  |
| 127590_PFF0 | XP_002614044.1 | GETC01080577.1 | JT854288.1 | O00468.5 |  |  | NtA |  | NtA |
|  |  |  |  |  | Kazal_2 | Kazal_2 | Kazal_2 |  | Kazal_2 |
|  |  |  |  |  | Kazal_2 | Kazal_2 | Kazal_2 |  | Kazal_2 |
|  |  |  |  |  | Kazal_2 | Kazal_2 | Kazal_2 |  | Kazal_2 |
|  |  |  |  |  | SEA | SEA | SEA |  |  |
|  |  |  |  |  | Kazal_1 | Kazal_2 | Kazal_1 |  | Kazal_1 |
|  |  |  |  |  | Kazal_2 | Kazal_2 | Kazal_2 |  | Kazal_2 |
|  |  |  |  |  | Kazal_2 | Kazal_2 | Kazal_2 |  | Kazal_2 |
|  |  |  |  |  | Kazal_2 | Kazal_1 | Kazal_1 |  | Kazal_1 |
|  |  |  |  |  | Kazal_2 | Kazal_1 | Kazal_2 |  | Kazal_2 |
|  |  |  |  |  | Laminin_EGF | Laminin_EGF | Laminin_EGF |  | Laminin_EGF |
|  |  |  |  |  | Laminin_EGF | Laminin_EGF | Laminin_EGF |  | Laminin_EGF |
|  |  |  |  |  |  | Kazal_2 | Kazal_2 |  | Kazal_2 |
|  |  |  |  |  |  |  |  |  | SEA |
|  |  |  |  |  |  | EGF | EGF |  | EGF |
|  |  |  |  |  |  | Laminin_G_1 | Laminin_G_1 |  | Laminin_G_1 |
|  |  |  |  |  |  | Laminin_G_1 | Laminin_G_1 |  | Laminin_G_1 |
|  |  |  |  |  |  | EGF | EGF |  | EGF |
|  |  |  |  |  |  | Laminin_G_1 | Laminin_G_1 | Laminin_G_1 | Laminin_G_1 |
|  |  |  |  |  |  |  |  |  |  |
|  |  |  | JT898669.1 | O00468.5 |  |  |  |  | NtA |
|  |  |  |  |  |  |  |  |  | Kazal_2 |
|  |  |  |  |  |  |  |  | Kazal_2 | Kazal_2 |
|  |  |  |  |  |  |  |  | Kazal_2 | Kazal_2 |
|  |  |  |  |  |  |  |  | SEA |  |
|  |  |  |  |  |  |  |  |  | Kazal_1 |
|  |  |  |  |  |  |  |  |  | Kazal_2 |
|  |  |  |  |  |  |  |  |  | Kazal_2 |
|  |  |  |  |  |  |  |  |  | Kazal_1 |
|  |  |  |  |  |  |  |  |  | Kazal_2 |
|  |  |  |  |  |  |  |  |  | Laminin_EGF |
|  |  |  |  |  |  |  |  |  | Laminin_EGF |
|  |  |  |  |  |  |  |  |  | Kazal_2 |
|  |  |  |  |  |  |  |  |  | SEA |
|  |  |  |  |  |  |  |  |  | EGF |
|  |  |  |  |  |  |  |  |  | Laminin_G_1 |
|  |  |  |  |  |  |  |  |  | Laminin_G_1 |
|  |  |  |  |  |  |  |  |  | EGF |
|  |  |  |  |  |  |  |  |  | Laminin_G_1 |
|  |  |  |  |  |  |  |  |  |  |
|  |  |  | JT855415.1 | O00468.5 |  |  |  |  | NtA |
|  |  |  |  |  |  |  |  |  | Kazal_2 |
|  |  |  |  |  |  |  |  |  | Kazal_2 |
|  |  |  |  |  |  |  |  |  | Kazal_2 |
|  |  |  |  |  |  |  |  |  |  |
|  |  |  |  |  |  |  |  | Kazal_1 | Kazal_1 |
|  |  |  |  |  |  |  |  | Kazal_2 | Kazal_2 |
|  |  |  |  |  |  |  |  | Kazal_2 | Kazal_2 |
|  |  |  |  |  |  |  |  | Kazal_2 | Kazal_1 |
|  |  |  |  |  |  |  |  |  | Kazal_2 |
|  |  |  |  |  |  |  |  |  | Laminin_EGF |
|  |  |  |  |  |  |  |  |  | Laminin_EGF |
|  |  |  |  |  |  |  |  |  | Kazal_2 |
|  |  |  |  |  |  |  |  |  | SEA |
|  |  |  |  |  |  |  |  |  | EGF |
|  |  |  |  |  |  |  |  |  | Laminin_G_1 |
|  |  |  |  |  |  |  |  |  | Laminin_G_1 |
|  |  |  |  |  |  |  |  |  | EGF |
|  |  |  |  |  |  |  |  |  | Laminin_G_1 |
|  |  |  |  |  |  |  |  |  |  |
| 127600_PFF0 | XP_002614044.1 | GETC01080577.1 | JT855415.1 | O00468.5 |  |  | NtA |  | NtA |
|  |  |  |  |  |  | Kazal_2 | Kazal_2 |  | Kazal_2 |
|  |  |  |  |  |  | Kazal_2 | Kazal_2 |  | Kazal_2 |
|  |  |  |  |  |  | Kazal_2 | Kazal_2 |  | Kazal_2 |
|  |  |  |  |  |  | SEA | SEA |  |  |
|  |  |  |  |  |  | Kazal_2 | Kazal_1 | Kazal_1 | Kazal_1 |
|  |  |  |  |  |  | Kazal_2 | Kazal_2 | Kazal_2 | Kazal_2 |
|  |  |  |  |  |  | Kazal_2 | Kazal_2 | Kazal_2 | Kazal_2 |
|  |  |  |  |  |  | Kazal_1 | Kazal_1 | Kazal_2 | Kazal_1 |
|  |  |  |  |  |  | Kazal_1 | Kazal_2 |  | Kazal_2 |
|  |  |  |  |  |  | Laminin_EGF | Laminin_EGF |  | Laminin_EGF |
|  |  |  |  |  |  | Laminin_EGF | Laminin_EGF |  | Laminin_EGF |
|  |  |  |  |  | Kazal_2 | Kazal_2 | Kazal_2 |  | Kazal_2 |
|  |  |  |  |  |  |  |  |  | SEA |
|  |  |  |  |  | EGF | EGF | EGF |  | EGF |
|  |  |  |  |  | Laminin_G_1 | Laminin_G_1 | Laminin_G_1 |  | Laminin_G_1 |
|  |  |  |  |  | Laminin_G_1 | Laminin_G_1 | Laminin_G_1 |  | Laminin_G_1 |
|  |  |  |  |  | EGF | EGF | EGF |  | EGF |
|  |  |  |  |  | Laminin_G_1 | Laminin_G_1 | Laminin_G_1 |  | Laminin_G_1 |
|  |  |  |  |  |  |  |  |  |  |
| 128050_PFF0 | XP_002603923.1 | GESY01067694.1 | JT866396.1 | P13671.3 | TSP_1 | TSP_1 | TSP_1 |  | TSP_1 |
|  |  |  |  |  |  | TSP_1 | TSP_1 |  | TSP_1 |
|  |  |  |  |  | Ldl_recept_a | Ldl_recept_a | Ldl_recept_a |  | Ldl_recept_a |
|  |  |  |  |  | MACPF |  |  |  |  |
|  |  |  |  |  | MACPF | MACPF | MACPF | MACPF | MACPF |
|  |  |  |  |  | TSP_1 | TSP_1 | TSP_1 | TSP_1 | TSP_1 |
|  |  |  |  |  | Ephrin_rec_like | Ephrin_rec_like | Ephrin_rec_like | Ephrin_rec_like |  |
|  |  |  |  |  |  |  |  |  | Sushi |
|  |  |  |  |  |  |  |  |  | Sushi |
|  |  |  |  |  |  |  |  |  |  |
| 133320_PFF0 | XP_002610402.1 | GETC01023426.1 |  | O08762.1 | SRCR | SRCR |  |  |  |
|  |  |  |  |  | Kringle | Kringle | Kringle |  | Kringle |
|  |  |  |  |  | SRCR | SRCR | SRCR |  | SRCR |
|  |  |  |  |  |  |  |  |  | SRCR |
|  |  |  |  |  |  |  |  |  | SRCR |
|  |  |  |  |  | Trypsin | Trypsin |  |  | Trypsin |
|  |  |  |  |  |  |  |  |  |  |
|  |  | GETC01023424.1 |  | O08762.1 |  |  |  |  |  |
|  |  |  |  |  |  |  |  |  | Kringle |
|  |  |  |  |  |  |  |  |  | SRCR |
|  |  |  |  |  |  |  |  |  | SRCR |
|  |  |  |  |  |  |  |  |  | SRCR |
|  |  |  |  |  |  |  | Trypsin |  | Trypsin |
|  |  |  |  |  |  |  |  |  |  |
| 135460_PFF0 | XP_002605157.1 | GESY01038239.1 | JT854244.1 | Q13467.2 | Fz | Fz | Fz | Fz | Fz |
|  |  |  |  |  | Frizzled | Frizzled | Frizzled | Frizzled | Frizzled |
|  |  |  |  |  |  |  |  |  |  |
| 144220_PFF0 | XP_002591459.1 | GETC01077238.1 | JT893228.1 | Q6QI06.2 | RICTOR_N | RICTOR_N | RICTOR_N | RICTOR_N | RICTOR_N |
|  |  |  |  |  | RICTOR_M | RICTOR_M | RICTOR_M |  | RICTOR_M |
|  |  |  |  |  | RasGEF_N_2 | RasGEF_N_2 | RasGEF_N_2 |  | RasGEF_N_2 |
|  |  |  |  |  | RICTOR_V | RICTOR_V | RICTOR_V |  | RICTOR_V |
|  |  |  |  |  |  |  |  |  | RICTOR_phospho |
|  |  |  |  |  |  |  |  |  |  |
| 152200_PFF0 | XP_002585927.1 | GESY01070048.1 | JT875956.1 | Q80U49.2 | FHA |  | FHA |  | FHA |
|  |  |  |  |  | CEP170_C |  | CEP170_C | CEP170_C | CEP170_C |
|  |  |  |  |  |  |  |  |  |  |
|  |  |  | JT885945.1 | Q80U49.2 |  |  |  | FHA | FHA |
|  |  |  |  |  |  |  |  |  | CEP170_C |
|  |  |  |  |  |  |  |  |  |  |
| 153460_PFF0 |  | GESY01070010.1 | JT878923.1 | O75762.3 |  |  |  |  | Ank |
|  |  |  |  |  |  |  |  |  | Ank |
|  |  |  |  |  |  |  |  |  | Ank |
|  |  |  |  |  | Ank |  | Ank |  | Ank |
|  |  |  |  |  | Ank |  | Ank |  | Ank |
|  |  |  |  |  | Ank |  | Ank |  | Ank |
|  |  |  |  |  | Ion_tran |  | Ion_tran | Ion_tran | Ion_tran |
|  |  |  |  |  |  |  |  |  |  |
| 157010_PFF0 | XP_002611039.1 |  |  | Q96PH1.1 | EF-hand_7 | EF-hand_7 |  |  | EF-hand_6 |
|  |  |  |  |  | Ferric_reduct | Ferric_reduct |  |  | Ferric_reduct |
|  |  |  |  |  | FAD_binding_8 |  |  |  | FAD_binding_8 |
|  |  |  |  |  | NAD_binding_6 | NAD_binding_6 |  |  | NAD_binding_6 |
|  |  |  |  |  |  |  |  |  |  |
| 157070_PFF0 | XP_002611055.1 | GETC01124911.1 | JT858939.1 | Q5T447.1 | ANAPC10 | ANAPC10 | ANAPC10 | ANAPC10 | ANAPC10 |
|  |  |  |  |  | HECT | HECT | HECT |  | HECT |
|  |  |  |  |  |  |  |  |  |  |
| 158710_PFF0 | XP_002600118.1 | GETC01124215.1 | JT885014.1 | P26640.4 |  |  |  |  | GST_C |
|  |  |  |  |  | tRNA-synt_1 | tRNA-synt_1 | tRNA-synt_1 |  | tRNA-synt_1 |
|  |  |  |  |  | Anticodon_1 | Anticodon_1 | Anticodon_1 | Anticodon_1 | Anticodon_1 |
|  |  |  |  |  |  |  |  |  |  |
|  |  |  | JT892791.1 | P26640.4 |  |  |  |  | GST_C |
|  |  |  |  |  |  |  |  | tRNA-synt_1 | tRNA-synt_1 |
|  |  |  |  |  |  |  |  |  | Anticodon_1 |
|  |  |  |  |  |  |  |  |  |  |
| 158830_PFF0 | XP_002600149.1 | GESY01060647.1 | JT873876.1 | P29268.3 | IGFBP | IGFBP | IGFBP |  | IGFBP |
|  |  |  |  |  | VWC | VWC | VWC |  | VWC |
|  |  |  |  |  | TSP_1 | TSP_1 | TSP_1 |  | TSP_1 |
|  |  |  |  |  | Cys_knot | Cys_knot | Cys_knot | Cys_knot | Cys_knot |
|  |  |  |  |  |  |  |  |  |  |
| 160400_PFF0 | XP_002601973.1 | GETC01134049.1 | JT869399.1 | P09055.1 | PSI_integrin | PSI_integrin | PSI_integrin |  | PSI_integrin |
|  |  |  |  |  | Integrin_beta | Integrin_beta | Integrin_beta |  | Integrin_beta |
|  |  |  |  |  | EGF_2 | EGF_2 | EGF_2 |  | EGF_2 |
|  |  |  |  |  | Integrin_B_tail | Integrin_B_tail | Integrin_B_tail | Integrin_B_tail | Integrin_B_tail |
|  |  |  |  |  | Integrin_b_cyt | Integrin_b_cyt | Integrin_b_cyt | Integrin_b_cyt | Integrin_b_cyt |
|  |  |  |  |  |  |  |  |  |  |
|  |  |  | JT902700.1 | P09055.1 |  |  |  |  | PSI_integrin |
|  |  |  |  |  |  |  |  |  | Integrin_beta |
|  |  |  |  |  |  |  |  | EGF_2 | EGF_2 |
|  |  |  |  |  |  |  |  |  | Integrin_B_tail |
|  |  |  |  |  |  |  |  |  | Integrin_b_cyt |
|  |  |  |  |  |  |  |  |  |  |
|  |  |  | JT865833.1 | P09055.1 |  |  |  |  | PSI_integrin |
|  |  |  |  |  |  |  |  | Integrin_beta | Integrin_beta |
|  |  |  |  |  |  |  |  |  | EGF_2 |
|  |  |  |  |  |  |  |  |  | Integrin_B_tail |
|  |  |  |  |  |  |  |  |  | Integrin_b_cyt |
|  |  |  |  |  |  |  |  |  |  |
| 161590_PFF0 | XP_002592408.1 | GESY01089392.1 | JT892500.1; JT866927.1;  JT864645.1; JT879667.1 | Q9Y4G6.4 |  |  | FERM_f0 |  | FERM_f0 |
|  |  |  |  |  |  |  | FERM_N |  | FERM_N |
|  |  |  |  |  | FERM_M |  | FERM_M |  | FERM_M |
|  |  |  |  |  | IRS |  | IRS |  | IRS |
|  |  |  |  |  | Talin_middle |  | Talin_middle |  | Talin_middle |
|  |  |  |  |  | I_LWEQ |  | I_LWEQ |  | I_LWEQ |
|  |  |  |  |  | VBS |  | VBS |  | VBS |
|  |  |  |  |  | VBS | VBS | VBS |  | VBS |
|  |  |  |  |  | I_LWEQ | I_LWEQ | I_LWEQ |  | I_LWEQ |
|  |  |  |  |  |  |  |  |  |  |
|  | XP_002592411.1 |  |  | Q9Y4G6.4 |  |  |  |  | FERM_f0 |
|  |  |  |  |  |  |  |  |  | FERM_N |
|  |  |  |  |  |  | FERM_M |  |  | FERM_M |
|  |  |  |  |  |  | IRS |  |  | IRS |
|  |  |  |  |  |  | Talin_middle |  |  | Talin_middle |
|  |  |  |  |  |  |  |  |  | I_LWEQ |
|  |  |  |  |  |  |  |  |  | VBS |
|  |  |  |  |  |  |  |  |  | VBS |
|  |  |  |  |  |  |  |  |  | I_LWEQ |
|  |  |  |  |  |  |  |  |  |  |
|  | XP_002592410.1 |  |  | Q9Y4G6.4 |  |  |  |  | FERM_f0 |
|  |  |  |  |  |  |  |  |  | FERM_N |
|  |  |  |  |  |  |  |  |  | FERM_M |
|  |  |  |  |  |  |  |  |  | IRS |
|  |  |  |  |  |  |  |  |  | Talin_middle |
|  |  |  |  |  |  | I_LWEQ |  |  | I_LWEQ |
|  |  |  |  |  |  | VBS |  |  | VBS |
|  |  |  |  |  |  |  |  |  | VBS |
|  |  |  |  |  |  |  |  |  | I_LWEQ |
|  |  |  |  |  |  |  |  |  |  |
|  |  |  | JT897439.1; JT855843.1 | Q9Y4G6.4 |  |  |  |  | FERM_f0 |
|  |  |  |  |  |  |  |  |  | FERM_N |
|  |  |  |  |  |  |  |  |  | FERM_M |
|  |  |  |  |  |  |  |  |  | IRS |
|  |  |  |  |  |  |  |  |  | Talin_middle |
|  |  |  |  |  |  |  |  |  | I_LWEQ |
|  |  |  |  |  |  |  |  |  | VBS |
|  |  |  |  |  |  |  |  |  | VBS |
|  |  |  |  |  |  |  |  | I_LWEQ | I_LWEQ |
|  |  |  |  |  |  |  |  |  |  |
|  |  |  | JT862406.1 | Q9Y4G6.4 |  |  |  | FERM_f0 | FERM_f0 |
|  |  |  |  |  |  |  |  |  | FERM_N |
|  |  |  |  |  |  |  |  |  | FERM_M |
|  |  |  |  |  |  |  |  |  | IRS |
|  |  |  |  |  |  |  |  |  | Talin_middle |
|  |  |  |  |  |  |  |  |  | I_LWEQ |
|  |  |  |  |  |  |  |  |  | VBS |
|  |  |  |  |  |  |  |  |  | VBS |
|  |  |  |  |  |  |  |  |  | I_LWEQ |
|  |  |  |  |  |  |  |  |  |  |
| 172520_PRF0 | XP_002611318.1 | GESY01038187.1 | JT882240.1 | Q96HF1.2 | Fz | Fz | Fz | Fz | Fz |
|  |  |  |  |  | NTR | NTR | NTR | NTR | NTR |
|  |  |  |  |  |  |  |  |  |  |
| 185920_PRF0 | XP_002606276.1 | GETC01050291.1 | JT892422.1 | P35419.1 | An_peroxidase | An_peroxidase | An_peroxidase | An_peroxidase | An_peroxidase |
|  |  |  |  |  |  | Sushi | Sushi |  | Sushi |
|  |  |  |  |  | EGF_CA | EGF_CA | EGF_CA |  | EGF_CA |
|  |  |  |  |  |  |  |  |  |  |
| 186040_PRF0 | XP_002606276.1 | GETC01050291.1 | JT892422.1 | P35419.1 | An_peroxidase | An_peroxidase | An_peroxidase | An_peroxidase | An_peroxidase |
|  |  |  |  |  | Sushi | Sushi | Sushi |  | Sushi |
|  |  |  |  |  | EGF_CA | EGF_CA | EGF_CA |  | EGF_CA |
|  |  |  |  |  |  |  |  |  |  |
| 189350_PRF0 | XP_002596820.1 | GETC01096834.1 | JT869040.1 | Q8CCP0.2 | FbpA | FbpA | FbpA |  | FbpA |
|  |  |  |  |  | DUF814 |  | DUF814 | DUF814 | DUF814 |
|  |  |  |  |  | DUF3441 |  |  | DUF3441 | DUF3441 |
|  |  |  |  |  |  |  |  |  |  |
|  |  | GETC01128145.1 |  | Q8CCP0.2 |  |  |  |  | FbpA |
|  |  |  |  |  |  |  |  |  | DUF814 |
|  |  |  |  |  |  |  | DUF3441 |  | DUF3441 |
|  |  |  |  |  |  |  |  |  |  |
| 192660_PRF0 | XP_002586029 | GETC01134001.1 | JT898480.1 | P22314.3 | ThiF | ThiF | ThiF | ThiF | ThiF |
|  |  |  |  |  | E1_FCCH | E1_FCCH | E1_FCCH | E1_FCCH | E1_FCCH |
|  |  |  |  |  | E1_4HB | E1_4HB | E1_4HB | E1_4HB | E1_4HB |
|  |  |  |  |  | ThiF | ThiF | ThiF | ThiF | ThiF |
|  |  |  |  |  | UBA_e1_thiolCys | UBA_e1_thiolCys | UBA_e1_thiolCys | UBA_e1_thiolCys | UBA_e1_thiolCys |
|  |  |  |  |  | E1_UFD | E1_UFD | E1_UFD | E1_UFD | E1_UFD |
|  |  |  |  |  |  |  |  |  |  |
| 196330_PRF0 | XP_002594642.1 | GETC01082826.1 | JT867825.1 | Q9EQJ0.1 | Ion_trans | Ion_trans | Ion_trans |  | Ion_trans |
|  |  |  |  |  | Ion_trans | Ion_trans | Ion_trans | Ion_trans | Ion_trans |
|  |  |  |  |  | Ion_trans |  |  |  |  |
|  |  |  |  |  | Ion_trans |  |  |  |  |
|  |  |  |  |  |  |  |  |  |  |
| 207610_PRF0 | XP_002610947.1 | GETC01064561.1 | JT881914.1 | Q96S42.2 | TGFb_propeptide | TGFb_propeptide | TGFb_propeptide | TGFb_propeptide | TGFb_propeptide |
|  |  |  |  |  | TGF_beta | TGF_beta | TGF_beta |  | TGF_beta |
|  |  |  |  |  |  |  |  |  |  |
|  |  |  | JT871298.1 | Q96S42.2 |  |  |  |  | TGFb_propeptide |
|  |  |  |  |  |  |  |  | TGF_beta | TGF_beta |
|  |  |  |  |  |  |  |  |  |  |
| 208940_PRF0 | XP_002607923.1 | GETC01020779.1 | JT853358.1 | P16066.1 | ANF_receptor |  |  |  | ANF_receptor |
|  |  |  |  |  | Pkinase_Tyr | Pkinase_Tyr |  |  | Pkinase_Tyr |
|  |  |  |  |  | Guanylate_cyc | Guanylate_cyc | Guanylate_cyc | Guanylate_cyc | Guanylate_cyc |
|  |  |  |  |  |  |  |  |  |  |
| 209230_PRF0 | XP_002610589.1 |  |  | P54131.1 | Neur_chan_LBD | Neur_chan_LBD |  |  | Neur_chan_LBD |
|  |  |  |  |  | Neur_chan_memb | Neur_chan_memb |  |  | Neur_chan_memb |
|  |  |  |  |  | Neur_chan_LBD |  |  |  |  |
|  |  |  |  |  | Neur_chan_memb |  |  |  |  |
|  |  |  |  |  |  |  |  |  |  |
| 211270_PRF0 | XP_002591619.1 | GETC01129814.1 | JT861679.1 | Q15057.3 | BAR_3 | BAR_3 | BAR_3 | BAR_3 | BAR_3 |
|  |  |  |  |  | PH | PH | PH |  | PH |
|  |  |  |  |  | ArfGap | ArfGap | ArfGap |  | ArfGap |
|  |  |  |  |  | Ank_2 | Ank_2 | Ank_2 |  | Ank_2 |
|  |  |  |  |  |  |  |  |  |  |
|  |  |  | JT867735.1 | Q15057.3 |  |  |  |  | BAR_3 |
|  |  |  |  |  |  |  |  |  | PH |
|  |  |  |  |  |  |  |  |  | ArfGap |
|  |  |  |  |  |  |  |  | Ank_2 | Ank_2 |
|  |  |  |  |  |  |  |  |  |  |
| 215230_PRF0 | XP_002589217.1  XP_002589229.1 | GETC01106794.1 | JT879834.1 | Q9UKY4.2 | PMT | PMT | PMT | PMT | PMT |
|  |  |  |  |  | MIR | MIR | MIR | MIR | MIR |
|  |  |  |  |  | PMT_4TMC | PMT_4TMC | PMT_4TMC |  | PMT_4TMC |
|  |  |  |  |  |  |  |  |  |  |
|  |  |  | JT884748.1 | Q9UKY4.2 |  |  |  |  | PMT |
|  |  |  |  |  |  |  |  |  | MIR |
|  |  |  |  |  |  |  |  | PMT_4TMC | PMT_4TMC |
|  |  |  |  |  |  |  |  |  |  |
| 222490_PRF0 | XP_002598311.1 | GETC01111356.1 | JT858521.1 | Q24K09.1 | DMAP_binding |  | DMAP_binding |  | DMAP_binding |
|  |  |  |  |  | DNMT1-RFD | DNMT1-RFD | DNMT1-RFD |  | DNMT1-RFD |
|  |  |  |  |  | zf-CXXC | zf-CXXC | zf-CXXC |  | zf-CXXC |
|  |  |  |  |  | BAH | BAH | BAH |  | BAH |
|  |  |  |  |  | BAH | BAH | BAH | BAH | BAH |
|  |  |  |  |  | DNA_methylase | DNA_methylase | DNA_methylase | DNA_methylase | DNA_methylase |
|  |  |  |  |  |  |  |  |  |  |
|  |  |  | JT854208.1 | Q24K09.1 |  |  |  | DMAP_binding | DMAP_binding |
|  |  |  |  |  |  |  |  | DNMT1-RFD | DNMT1-RFD |
|  |  |  |  |  |  |  |  | zf-CXXC | zf-CXXC |
|  |  |  |  |  |  |  |  | BAH | BAH |
|  |  |  |  |  |  |  |  |  | BAH |
|  |  |  |  |  |  |  |  |  | DNA_methylase |
|  |  |  |  |  |  |  |  |  |  |
| 223320_PRF0 | XP_002601301.1 | GETC01005797.1 |  | Q8TE56.2 | Pep_M12B_propep | Pep_M12B_propep |  |  | Pep_M12B_propep |
|  |  |  |  |  | Reprolysin | Reprolysin |  |  | Reprolysin |
|  |  |  |  |  | TSP_1 | TSP_1 |  |  | TSP_1 |
|  |  |  |  |  | ADAM_spacer1 | ADAM_spacer1 | ADAM_spacer1 |  | ADAM_spacer1 |
|  |  |  |  |  | TSP_1 | TSP_1 |  |  | TSP_1 |
|  |  |  |  |  | TSP_1 | TSP_1 |  |  | TSP_1 |
|  |  |  |  |  | TSP_1 | TSP_1 |  |  | TSP_1 |
|  |  |  |  |  | TSP_1 | TSP_1 |  |  |  |
|  |  |  |  |  |  | TSP_1 |  |  |  |
|  |  |  |  |  |  | TSP_1 |  |  |  |
|  |  |  |  |  |  | TSP_1 |  |  |  |
|  |  |  |  |  |  |  |  |  |  |
|  |  | GETC01033854.1 |  | Q8TE56.2 |  |  |  |  | Pep_M12B_propep |
|  |  |  |  |  |  |  | Reprolysin |  | Reprolysin |
|  |  |  |  |  |  |  |  |  | TSP_1 |
|  |  |  |  |  |  |  |  |  | ADAM_spacer1 |
|  |  |  |  |  |  |  |  |  | TSP_1 |
|  |  |  |  |  |  |  |  |  | TSP_1 |
|  |  |  |  |  |  |  |  |  | TSP_1 |
|  |  |  |  |  |  |  |  |  |  |
|  |  |  |  |  |  |  |  |  |  |
|  |  |  |  |  |  |  |  |  |  |
|  |  |  |  |  |  |  |  |  |  |
|  |  |  |  |  |  |  |  |  |  |
|  |  | GETC01144856.1 |  | Q8TE56.2 |  |  |  |  | Pep_M12B_propep |
|  |  |  |  |  |  |  |  |  | Reprolysin |
|  |  |  |  |  |  |  |  |  | TSP_1 |
|  |  |  |  |  |  |  |  |  | ADAM_spacer1 |
|  |  |  |  |  |  |  | TSP_1 |  | TSP_1 |
|  |  |  |  |  |  |  | TSP_1 |  | TSP_1 |
|  |  |  |  |  |  |  |  |  | TSP_1 |
|  |  |  |  |  |  |  |  |  |  |
|  |  |  |  |  |  |  |  |  |  |
|  |  |  |  |  |  |  |  |  |  |
|  |  |  |  |  |  |  |  |  |  |
|  |  |  |  |  |  |  |  |  |  |
| 227800_PRF0 | XP_002608349.1 | GESY01076858.1 | JT871508.1 | Q9Z175.2 |  |  | SRCR |  | SRCR |
|  |  |  |  |  | SRCR | SRCR | SRCR |  | SRCR |
|  |  |  |  |  | SRCR | SRCR | SRCR | SRCR | SRCR |
|  |  |  |  |  | SRCR | SRCR | SRCR | SRCR | SRCR |
|  |  |  |  |  | Lysyl_oxidase | Lysyl_oxidase | Lysyl_oxidase | Lysyl_oxidase | Lysyl_oxidase |
|  |  |  |  |  |  | SRCR |  |  |  |
|  |  |  |  |  |  | Lysyl_oxidase |  |  |  |
|  |  |  |  |  |  | Lysyl_oxidase |  |  |  |
|  |  |  |  |  |  |  |  |  |  |
| 237250_PRF0 | XP_002612866.1 | GETC01135602.1 | JT901966.1 | Q00685.1 |  |  | A2M_N |  | A2M_N |
|  |  |  |  |  |  |  | A2M_N_2 |  | A2M_N_2 |
|  |  |  |  |  |  | ANATO | ANATO |  | ANATO |
|  |  |  |  |  |  | A2M | A2M |  | A2M |
|  |  |  |  |  |  | Thiol-ester_cl | Thiol-ester_cl |  | Thiol-ester_cl |
|  |  |  |  |  |  | A2M_comp | A2M_comp |  | A2M_comp |
|  |  |  |  |  | A2M_recep | A2M_recep | A2M_recep |  | A2M_recep |
|  |  |  |  |  | NTR | NTR | NTR | NTR | NTR |
|  |  |  |  |  |  |  |  |  |  |
| 237260_PRF0 | XP_002612866.1 | GETC01135602.1 | JT901966.1 | Q00685.1 | A2M_N |  | A2M_N |  | A2M_N |
|  |  |  |  |  | A2M_N_2 |  | A2M_N_2 |  | A2M_N_2 |
|  |  |  |  |  | ANATO | ANATO | ANATO |  | ANATO |
|  |  |  |  |  | A2M | A2M | A2M |  | A2M |
|  |  |  |  |  | Thiol-ester_cl | Thiol-ester_cl | Thiol-ester_cl |  | Thiol-ester_cl |
|  |  |  |  |  | A2M_comp | A2M_comp | A2M_comp |  | A2M_comp |
|  |  |  |  |  |  | A2M_recep | A2M_recep |  | A2M_recep |
|  |  |  |  |  |  | NTR | NTR |  | NTR |
|  |  |  |  |  |  |  |  |  |  |
|  | AF391289_5 |  |  | Q00685.1 |  | A2M_N |  |  | A2M_N |
|  |  |  |  |  |  | A2M_N_2 |  |  | A2M_N_2 |
|  |  |  |  |  |  | ANATO |  |  | ANATO |
|  |  |  |  |  |  | A2M |  |  | A2M |
|  |  |  |  |  |  |  |  |  | Thiol-ester_cl |
|  |  |  |  |  |  |  |  |  | A2M_comp |
|  |  |  |  |  |  |  |  |  | A2M_recep |
|  |  |  |  |  |  |  |  |  | NTR |
|  |  |  |  |  |  |  |  |  |  |
| 246010_PRF0 | XP_002613969.1 | GETC01016608.1 | JT878297.1 | Q99542.1 | PG_binding_1 | PG_binding_1 |  |  | PG_binding_1 |
|  |  |  |  |  | Peptidase_M10 | Peptidase_M10 | Peptidase_M10 |  | Peptidase_M10 |
|  |  |  |  |  | Hemopexin | Hemopexin |  | Hemopexin | Hemopexin |
|  |  |  |  |  | Hemopexin | Hemopexin |  | Hemopexin | Hemopexin |
|  |  |  |  |  | Hemopexin | Hemopexin |  |  | Hemopexin |
|  |  |  |  |  | Hemopexin | Hemopexin |  |  | Hemopexin |
|  |  |  |  |  |  |  |  |  |  |
|  |  | GETC01070986.1 |  | Q99542.1 |  |  |  |  | PG_binding_1 |
|  |  |  |  |  |  |  |  |  | Peptidase_M10 |
|  |  |  |  |  |  |  | Hemopexin |  | Hemopexin |
|  |  |  |  |  |  |  | Hemopexin |  | Hemopexin |
|  |  |  |  |  |  |  | Hemopexin |  | Hemopexin |
|  |  |  |  |  |  |  | Hemopexin |  | Hemopexin |
|  |  |  |  |  |  |  |  |  |  |
| 247370_PRF0 |  | GETC01129892.1 | JT858095.1 | P34925.2 | WIF |  | WIF | WIF | WIF |
|  |  |  |  |  | Pkinase_Tyr |  | Pkinase_Tyr |  | Pkinase_Tyr |
|  |  |  |  |  |  |  |  |  |  |
|  |  |  | JT874093.1 | P34925.2 |  |  |  |  | WIF |
|  |  |  |  |  |  |  |  | Pkinase_Tyr | Pkinase_Tyr |
|  |  |  |  |  |  |  |  |  |  |
| 248080_PRF0 | XP_002591824.1 | GETC01076328.1 |  | P06681.2 | EGF_3 |  | EGF_3 |  | EGF_3 |
|  |  |  |  |  | Sushi | Sushi | Sushi |  | Sushi |
|  |  |  |  |  | Sushi | Sushi | Sushi |  | Sushi |
|  |  |  |  |  | VWA | VWA | VWA |  | VWA |
|  |  |  |  |  | Trypsin | Trypsin | Trypsin |  | Trypsin |
|  |  |  |  |  |  |  |  |  |  |
| 253950_PRF0 | XP_002610496.1 | GETC01133873.1 | JT900403.1 | Q07890.2 | Histone | Histone | Histone |  | Histone |
|  |  |  |  |  | RhoGEF | RhoGEF | RhoGEF |  | RhoGEF |
|  |  |  |  |  | PH | PH | PH | PH | PH |
|  |  |  |  |  | RasGEF_N | RasGEF_N | RasGEF_N | RasGEF_N | RasGEF_N |
|  |  |  |  |  | RasGEF | RasGEF | RasGEF | RasGEF | RasGEF |
|  |  |  |  |  |  |  |  |  |  |
| 253960_PRF0 | XP_002610495.1 | GESY01082167.1 | JT886190.1 | Q15436.2 | zf-Sec23_Sec24 | zf-Sec23_Sec24 | zf-Sec23_Sec24 | zf-Sec23_Sec24 | zf-Sec23_Sec24 |
|  |  |  |  |  | Sec23_trunk | Sec23_trunk | Sec23_trunk | Sec23_trunk | Sec23_trunk |
|  |  |  |  |  | Sec23_BS | Sec23_BS | Sec23_BS |  | Sec23_BS |
|  |  |  |  |  | Sec23_helical | Sec23_helical | Sec23_helical |  | Sec23_helical |
|  |  |  |  |  | Gelsolin | Gelsolin | Gelsolin |  | Gelsolin |
|  |  |  |  |  |  |  |  |  |  |
|  |  |  | JT854459.1 | Q15436.2 |  |  |  |  | zf-Sec23_Sec24 |
|  |  |  |  |  |  |  |  | Sec23_trunk | Sec23_trunk |
|  |  |  |  |  |  |  |  | Sec23_BS | Sec23_BS |
|  |  |  |  |  |  |  |  | Sec23_helical | Sec23_helical |
|  |  |  |  |  |  |  |  | Gelsolin | Gelsolin |
|  |  |  |  |  |  |  |  |  |  |
| 254170_PRF0 | XP_002595526.1 | GESY01074936.1 | JT867227.1 | O60706.2 | ABC_membrane | ABC_membrane | ABC_membrane |  | ABC_membrane |
|  |  |  |  |  | ABC_tran | ABC_tran | ABC_tran | ABC_tran | ABC_tran |
|  |  |  |  |  | ABC_membrane | ABC_membrane | ABC_membrane |  | ABC_membrane |
|  |  |  |  |  | ABC_tran | ABC_tran | ABC_tran |  | ABC_tran |
|  |  |  |  |  |  |  |  |  |  |
|  |  |  | JT888258.1 | O60706.2 |  |  |  |  | ABC_membrane |
|  |  |  |  |  |  |  |  |  | ABC_tran |
|  |  |  |  |  |  |  |  |  | ABC_membrane |
|  |  |  |  |  |  |  |  | ABC_tran | ABC_tran |
|  |  |  |  |  |  |  |  |  |  |
| 256520_PRF0 | B6MUN4.1 | GETC01084111.1 | JT893640.1 | Q5VVJ2.1 |  |  | Myb_DNA-binding | Myb_DNA-binding | Myb_DNA-binding |
|  |  |  |  |  |  | SWIRM | SWIRM |  | SWIRM |
|  |  |  |  |  | JAB | JAB | JAB |  | JAB |
|  |  |  |  |  |  |  |  |  |  |
| 256530_PRF0 | B6MUN4.1 | GETC01084111.1 | JT893640.1 | Q5VVJ2.1 | Myb_DNA-binding |  | Myb_DNA-binding | Myb_DNA-binding | Myb_DNA-binding |
|  |  |  |  |  | SWIRM | SWIRM | SWIRM |  | SWIRM |
|  |  |  |  |  |  | JAB | JAB |  | JAB |
|  |  |  |  |  |  |  |  |  |  |
| 257610_PRF0 | XP_002587133.1 | GETC01127440.1 | JT860571.1 | Q9UPR3.3 | EST1 | EST1 | EST1 |  | EST1 |
|  |  |  |  |  | EST1_DNA_bind | EST1_DNA_bind | EST1_DNA_bind | EST1_DNA_bind | EST1_DNA_bind |
|  |  |  |  |  | EST1_DNA_bind | EST1_DNA_bind | EST1_DNA_bind |  | EST1_DNA_bind |
|  |  |  |  |  | PIN_4 | PIN_4 | PIN_4 |  | PIN_4 |
|  |  |  |  |  |  |  |  |  |  |
| 258810_PRF0 | XP_002605408.1 | GESY01081071.1 | JT883826.1 | Q9BVH7.1 | Glyco_transf_29 | Glyco_transf_29 | Glyco_transf_29 | Glyco_transf_29 | Glyco_transf_29 |
|  |  |  |  |  | Glyco_transf_29 |  |  |  |  |
|  |  |  |  |  |  |  |  |  |  |
| 258870_PRF0 | XP_002595259.1 | GESY01087334.1 | JT854419.1 | P42229.1 | STAT_int |  | STAT_int |  | STAT_int |
|  |  |  |  |  | STAT_alpha | STAT_alpha | STAT_alpha | STAT_alpha | STAT_alpha |
|  |  |  |  |  | STAT_bind | STAT_bind | STAT_bind | STAT_bind | STAT_bind |
|  |  |  |  |  | SH2 | SH2 | SH2 | SH2 | SH2 |
|  |  |  |  |  |  |  |  |  |  |
|  |  |  | JT882393.1 | P42229.1 |  |  |  | STAT_int | STAT_int |
|  |  |  |  |  |  |  |  |  | STAT_alpha |
|  |  |  |  |  |  |  |  |  | STAT_bind |
|  |  |  |  |  |  |  |  |  | SH2 |
|  |  |  |  |  |  |  |  |  |  |
|  | XP_002585719.1 |  |  | P42229.1 |  | STAT_int |  |  | STAT_int |
|  |  |  |  |  |  |  |  |  | STAT_alpha |
|  |  |  |  |  |  |  |  |  | STAT_bind |
|  |  |  |  |  |  |  |  |  | SH2 |
|  |  |  |  |  |  |  |  |  |  |
|  | XP_002595260.1 |  |  | P42229.1 |  |  |  |  | STAT_int |
|  |  |  |  |  |  |  |  |  | STAT_alpha |
|  |  |  |  |  |  | STAT_bind |  |  | STAT_bind |
|  |  |  |  |  |  |  |  |  | SH2 |
|  |  |  |  |  |  |  |  |  |  |
|  | XP_002595256.1 |  |  | P42229.1 |  |  |  |  | STAT_int |
|  |  |  |  |  |  |  |  |  | STAT_alpha |
|  |  |  |  |  |  | STAT_bind |  |  | STAT_bind |
|  |  |  |  |  |  | SH2 |  |  | SH2 |
|  |  |  |  |  |  |  |  |  |  |
| 259020_PRF0 | XP_002600999.1 | GETC01117441.1 | JT857145.1 | Q6ZT07.2 | GRAM | GRAM | GRAM | No Pfam-A domain | GRAM |
|  |  |  |  |  | GRAM | GRAM | GRAM |  | GRAM |
|  |  |  |  |  | RabGAP-TBC | RabGAP-TBC | RabGAP-TBC |  | RabGAP-TBC |
|  |  |  |  |  |  |  |  |  |  |
| 272770_PRF0 | XP_002601715.1 | GESY01056426.1 |  | Q9BXT6.1 |  |  | S1-like |  |  |
|  |  |  |  |  |  |  | S1-like |  |  |
|  |  |  |  |  | AAA_11 | AAA_11 | AAA_11 |  | AAA_11 |
|  |  |  |  |  | AAA_11 | AAA_11 | AAA_11 |  | AAA_11 |
|  |  |  |  |  | AAA_12 | AAA_12 | AAA_12 |  | AAA_12 |
|  |  |  |  |  |  |  |  |  |  |
| 293630_PRF0 | XP_002613483.1 | GESY01088945.1 | JT860205.1; JT886810.1 | Q96BY7.5 | Chorein_N | Chorein_N | Chorein_N |  | Chorein_N |
|  |  |  |  |  | ATG2_CAD | ATG2_CAD | ATG2_CAD |  | ATG2_CAD |
|  |  |  |  |  | ATG_C | ATG_C | ATG_C |  | ATG_C |
|  |  |  |  |  |  |  |  |  |  |
| 293740_PRF0 | XP_002613924.1 | GESY01031847.1 | JT905168.1; JT881184.1 | Q5THJ4.2 |  |  | Chorein_N |  | Chorein_N |
|  |  |  |  |  |  | VPS13 | VPS13 |  | VPS13 |
|  |  |  |  |  |  | VPS13_mid_rpt |  |  | VPS13_mid_rpt |
|  |  |  |  |  |  | UBA |  |  | UBA |
|  |  |  |  |  | SHR-BD | SHR-BD |  |  | SHR-BD |
|  |  |  |  |  | VPS13_C | VPS13_C |  |  | VPS13_C |
|  |  |  |  |  |  |  |  |  |  |
|  |  | GESY01031849.1 |  | Q5THJ4.2 |  |  |  |  | Chorein_N |
|  |  |  |  |  |  |  | VPS13 |  | VPS13 |
|  |  |  |  |  |  |  | VPS13_mid_rpt |  | VPS13_mid_rpt |
|  |  |  |  |  |  |  |  |  | UBA |
|  |  |  |  |  |  |  |  |  | SHR-BD |
|  |  |  |  |  |  |  |  |  | VPS13_C |
|  |  |  |  |  |  |  |  |  |  |
|  |  | GESY01036158.1 |  | Q5THJ4.2 |  |  |  |  | Chorein_N |
|  |  |  |  |  |  |  |  |  | VPS13 |
|  |  |  |  |  |  |  |  |  | VPS13_mid_rpt |
|  |  |  |  |  |  |  |  |  | UBA |
|  |  |  |  |  |  |  |  |  | SHR-BD |
|  |  |  |  |  |  |  |  |  | VPS13_C |
|  |  |  |  |  |  |  |  |  |  |
|  |  | GETC01066591.1 |  | Q5THJ4.2 |  |  |  |  | Chorein_N |
|  |  |  |  |  |  |  |  |  | VPS13 |
|  |  |  |  |  |  |  |  |  | VPS13_mid_rpt |
|  |  |  |  |  |  |  | UBA |  | UBA |
|  |  |  |  |  |  |  |  |  | SHR-BD |
|  |  |  |  |  |  |  |  |  | VPS13_C |
|  |  |  |  |  |  |  |  |  |  |
|  |  | GESY01047751.1 |  | Q5THJ4.2 |  |  |  |  | Chorein_N |
|  |  |  |  |  |  |  |  |  | VPS13 |
|  |  |  |  |  |  |  |  |  | VPS13_mid_rpt |
|  |  |  |  |  |  |  |  |  | UBA |
|  |  |  |  |  |  |  | SHR-BD |  | SHR-BD |
|  |  |  |  |  |  |  |  |  | VPS13_C |
|  |  |  |  |  |  |  |  |  |  |
|  |  | GETC01099602.1 |  | Q5THJ4.2 |  |  |  |  | Chorein_N |
|  |  |  |  |  |  |  |  |  | VPS13 |
|  |  |  |  |  |  |  |  |  | VPS13_mid_rpt |
|  |  |  |  |  |  |  |  |  | UBA |
|  |  |  |  |  |  |  | SHR-BD |  | SHR-BD |
|  |  |  |  |  |  |  | VPS13_C |  | VPS13_C |
|  |  |  |  |  |  |  |  |  |  |
|  |  | GESY01047748.1 |  | Q5THJ4.2 |  |  |  |  | Chorein_N |
|  |  |  |  |  |  |  |  |  | VPS13 |
|  |  |  |  |  |  |  |  |  | VPS13_mid_rpt |
|  |  |  |  |  |  |  |  |  | UBA |
|  |  |  |  |  |  |  |  |  | SHR-BD |
|  |  |  |  |  |  |  | VPS13_C |  | VPS13_C |
|  |  |  |  |  |  |  |  |  |  |
| 209920_PRM0 | XP_002613924.1 | GESY01047748.1 | JT905168.1; JT881184.1 | Q5THJ4.2 |  |  |  |  | Chorein_N |
|  |  |  |  |  |  | VPS13 |  |  | VPS13 |
|  |  |  |  |  |  | VPS13_mid_rpt |  |  | VPS13_mid_rpt |
|  |  |  |  |  | UBA | UBA |  |  | UBA |
|  |  |  |  |  |  | SHR-BD |  |  | SHR-BD |
|  |  |  |  |  |  | VPS13_C | VPS13_C |  | VPS13_C |
|  |  |  |  |  |  |  |  |  |  |
| 209930_PRF0 | XP_002613924.1 | GESY01047748.1 | JT905168.1; JT881184.1 | Q5THJ4.2 |  |  |  |  | Chorein_N |
|  |  |  |  |  |  | VPS13 |  |  | VPS13 |
|  |  |  |  |  |  | VPS13_mid_rpt |  |  | VPS13_mid_rpt |
|  |  |  |  |  |  | UBA |  |  | UBA |
|  |  |  |  |  |  | SHR-BD |  |  | SHR-BD |
|  |  |  |  |  |  | VPS13_C | VPS13_C |  | VPS13_C |
|  |  |  |  |  |  |  |  |  |  |
| 209940_PRF0 | XP_002613924.1 | GETC01048876.1 |  | Q5THJ4.2 | Chorein_N |  | Chorein_N |  | Chorein_N |
|  |  |  |  |  | VPS13 | VPS13 | VPS13 |  | VPS13 |
|  |  |  |  |  | VPS13_mid_rpt | VPS13_mid_rpt |  |  | VPS13_mid_rpt |
|  |  |  |  |  |  | UBA |  |  | UBA |
|  |  |  |  |  |  | SHR-BD |  |  | SHR-BD |
|  |  |  |  |  |  | VPS13_C |  |  | VPS13_C |
|  |  |  |  |  |  |  |  |  |  |
|  | XP_002613923.1 |  |  | Q5THJ4.2 |  | Chorein_N |  |  | Chorein_N |
|  |  |  |  |  |  |  |  |  | VPS13 |
|  |  |  |  |  |  |  |  |  | VPS13_mid_rpt |
|  |  |  |  |  |  |  |  |  | UBA |
|  |  |  |  |  |  |  |  |  | SHR-BD |
|  |  |  |  |  |  |  |  |  | VPS13_C |
|  |  |  |  |  |  |  |  |  |  |
|  |  | GESY01031848.1 |  | Q5THJ4.2 |  |  |  |  | Chorein_N |
|  |  |  |  |  |  |  |  |  | VPS13 |
|  |  |  |  |  |  |  | VPS13_mid_rpt |  | VPS13_mid_rpt |
|  |  |  |  |  |  |  |  |  | UBA |
|  |  |  |  |  |  |  |  |  | SHR-BD |
|  |  |  |  |  |  |  |  |  | VPS13_C |
|  |  |  |  |  |  |  |  |  |  |
| 321850_PRF0 | XP_002585883.1 |  |  | Q13002.1 | ANF_receptor | ANF_receptor |  |  | ANF_receptor |
|  |  |  |  |  | Lig_chan-Glu_bd | Lig_chan-Glu_bd |  |  | Lig_chan-Glu_bd |
|  |  |  |  |  | Lig_chan | Lig_chan |  |  | Lig_chan |
|  |  |  |  |  |  |  |  |  |  |
| 319230_PRF0 | XP_002600703.1 | GETC01122700.1 | JT847968.1 | Q8WZA2.1 | cNMP_binding | cNMP_binding |  |  | cNMP_binding |
|  |  |  |  |  | DEP | DEP | DEP |  | DEP |
|  |  |  |  |  | cNMP_binding | cNMP_binding | cNMP_binding |  | cNMP_binding |
|  |  |  |  |  | RasGEF_N |  | RasGEF_N |  | RasGEF_N |
|  |  |  |  |  | RasGEF |  | RasGEF |  | RasGEF |
|  |  |  |  |  |  |  |  |  |  |
|  | XP_002600702.1 |  |  | Q8WZA2.1 |  |  |  |  | cNMP_binding |
|  |  |  |  |  |  |  |  |  | DEP |
|  |  |  |  |  |  |  |  |  | cNMP_binding |
|  |  |  |  |  |  |  |  |  | RasGEF_N |
|  |  |  |  |  |  | RasGEF |  |  | RasGEF |
|  |  |  |  |  |  |  |  |  |  |
| 325460_PRF0 | XP_002608790.1 | GESY01088299.1 | JT905598.1 | P53708.3 | FG-GAP | FG-GAP | FG-GAP |  | FG-GAP |
|  |  |  |  |  | FG-GAP | FG-GAP | FG-GAP |  | FG-GAP |
|  |  |  |  |  | FG-GAP | FG-GAP | FG-GAP |  | FG-GAP |
|  |  |  |  |  | FG-GAP | FG-GAP | FG-GAP |  | FG-GAP |
|  |  |  |  |  | FG-GAP | FG-GAP | FG-GAP |  | FG-GAP |
|  |  |  |  |  | FG-GAP | FG-GAP | FG-GAP |  | FG-GAP |
|  |  |  |  |  | FG-GAP | FG-GAP | FG-GAP |  | FG-GAP |
|  |  |  |  |  | Integrin_alpha2 | Integrin_alpha2 | Integrin_alpha2 | Integrin_alpha2 | Integrin_alpha2 |
|  |  |  |  |  | Integrin_alpha | Integrin_alpha | Integrin_alpha |  | Integrin_alpha |
|  |  |  |  |  |  |  |  |  |  |
|  |  |  | JT893300.1 | P53708.3 |  |  |  |  | FG-GAP |
|  |  |  |  |  |  |  |  |  | FG-GAP |
|  |  |  |  |  |  |  |  |  | FG-GAP |
|  |  |  |  |  |  |  |  |  | FG-GAP |
|  |  |  |  |  |  |  |  |  | FG-GAP |
|  |  |  |  |  |  |  |  |  | FG-GAP |
|  |  |  |  |  |  |  |  |  | FG-GAP |
|  |  |  |  |  |  |  |  | Integrin_alpha2 | Integrin_alpha2 |
|  |  |  |  |  |  |  |  | Integrin_alpha | Integrin_alpha |
|  |  |  |  |  |  |  |  |  |  |
| 320370_PRF0 | XP_002594515.1 |  | JT848714.1 | O15344.1 | zf-RING_UBOX | zf-RING_UBOX |  | zf-RING_UBOX | zf-RING_UBOX |
|  |  |  |  |  | zf-B_box | zf-B_box |  |  | zf-B_box |
|  |  |  |  |  | fn3 | fn3 |  |  | fn3 |
|  |  |  |  |  |  |  |  |  | PRY |
|  |  |  |  |  | SPRY |  |  |  | SPRY |
|  |  |  |  |  |  |  |  |  |  |
| 325780_PRF0 | XP_002602117.1 |  |  | Q10472.1 | Glycos_transf_2 | Glycos_transf_2 |  |  | Glycos_transf_2 |
|  |  |  |  |  | Ricin_B_lectin | Ricin_B_lectin |  |  | Ricin_B_lectin |
|  |  |  |  |  |  |  |  |  |  |
| 328450_PRF0 | XP_002607180.1 | GESY01032163.1 |  | Q8TEU8.1 |  | WAP |  |  | WAP |
|  |  |  |  |  |  | Kazal_2 |  |  | Kazal_2 |
|  |  |  |  |  |  | I-set | I-set |  | I-set |
|  |  |  |  |  |  | Kunitz_BPTI | Kunitz_BPTI |  | Kunitz_BPTI |
|  |  |  |  |  | Kunitz_BPTI | Kunitz_BPTI | Kunitz_BPTI |  | Kunitz_BPTI |
|  |  |  |  |  | NTR | NTR | NTR |  | NTR |
|  |  |  |  |  |  |  |  |  |  |
|  |  | GESY01032162.1 |  | Q8TEU8.1 |  |  |  |  | WAP |
|  |  |  |  |  |  |  | Kazal_2 |  | Kazal_2 |
|  |  |  |  |  |  |  | I-set |  | I-set |
|  |  |  |  |  |  |  |  |  | Kunitz_BPTI |
|  |  |  |  |  |  |  |  |  | Kunitz_BPTI |
|  |  |  |  |  |  |  |  |  | NTR |
|  |  |  |  |  |  |  |  |  |  |
|  |  | GESY01012772.1 |  | Q8TEU8.1 |  |  | WAP |  | WAP |
|  |  |  |  |  |  |  |  |  | Kazal_2 |
|  |  |  |  |  |  |  |  |  | I-set |
|  |  |  |  |  |  |  |  |  | Kunitz_BPTI |
|  |  |  |  |  |  |  |  |  | Kunitz_BPTI |
|  |  |  |  |  |  |  |  |  | NTR |
|  |  |  |  |  |  |  |  |  |  |
| 328460_PRF0 | XP_002607180.1 | GESY01032163.1 |  | Q8TEU8.1 | WAP | WAP |  |  | WAP |
|  |  |  |  |  | Kazal_2 | Kazal_2 |  |  | Kazal_2 |
|  |  |  |  |  | I-set | I-set | I-set |  | I-set |
|  |  |  |  |  | Kunitz_BPTI | Kunitz_BPTI | Kunitz_BPTI |  | Kunitz_BPTI |
|  |  |  |  |  | Kunitz_BPTI | Kunitz_BPTI | Kunitz_BPTI |  | Kunitz_BPTI |
|  |  |  |  |  |  | NTR | NTR |  | NTR |
|  |  |  |  |  |  |  |  |  |  |
| 328470_PRF0 | XP_002607185.1 | GESY01088448.1 | JT864843.1 | P33527.3 | ABC_membrane |  |  |  |  |
|  |  |  |  |  | ABC_tran |  |  |  |  |
|  |  |  |  |  | ABC_membrane | ABC_membrane | ABC_membrane |  | ABC_membrane |
|  |  |  |  |  | ABC_tran | ABC_tran | ABC_tran |  | ABC_tran |
|  |  |  |  |  |  | ABC_membrane |  |  |  |
|  |  |  |  |  |  | ABC_membrane |  |  |  |
|  |  |  |  |  |  | ABC_membrane |  |  |  |
|  |  |  |  |  |  | ABC_membrane |  |  |  |
|  |  |  |  |  | ABC_membrane | ABC_membrane | ABC_membrane | ABC_membrane | ABC_membrane |
|  |  |  |  |  | ABC_tran | ABC_tran | ABC_tran |  | ABC_tran |
|  |  |  |  |  | ABC_membrane |  |  |  |  |
|  |  |  |  |  | ABC_tran |  |  |  |  |
|  |  |  |  |  |  |  |  |  |  |
|  |  |  | JT904893.1 | P33527.3 |  |  |  |  | ABC_membrane |
|  |  |  |  |  |  |  |  |  | ABC_tran |
|  |  |  |  |  |  |  |  |  | ABC_membrane |
|  |  |  |  |  |  |  |  | ABC_tran | ABC_tran |
|  |  |  |  |  |  |  |  |  |  |
| 328810_PRF0 | XP_002592166.1 | GESY01056060.1 | JT855045.1 | Q8NEZ4.3 |  |  |  |  | zf-HC5HC2H |
|  |  |  |  |  |  |  |  |  | PHD |
|  |  |  |  |  |  |  |  |  | PHD |
|  |  |  |  |  | zf-HC5HC2H | zf-HC5HC2H | zf-HC5HC2H | zf-HC5HC2H | zf-HC5HC2H |
|  |  |  |  |  | FYRN | FYRN | FYRN | FYRN | FYRN |
|  |  |  |  |  | FYRC | FYRC | FYRC |  | FYRC |
|  |  |  |  |  | SET | SET | SET |  | SET |
|  |  |  |  |  |  |  |  |  |  |
|  |  |  | JT868966.1 | Q8NEZ4.3 |  |  |  |  | zf-HC5HC2H |
|  |  |  |  |  |  |  |  |  | PHD |
|  |  |  |  |  |  |  |  |  | PHD |
|  |  |  |  |  |  |  |  |  | zf-HC5HC2H |
|  |  |  |  |  |  |  |  |  | FYRN |
|  |  |  |  |  |  |  |  |  | FYRC |
|  |  |  |  |  |  |  |  | SET | SET |
|  |  |  |  |  |  |  |  |  |  |
| 328940_PRF0 | XP_002612925.1 | GETC01111835.1 | JT898789.1 | P78563.1 | dsrm |  | dsrm |  | dsrm |
|  |  |  |  |  | dsrm |  | dsrm |  | dsrm |
|  |  |  |  |  | A_deamin | A_deamin | A_deamin | A_deamin | A_deamin |
|  |  |  |  |  | A_deamin |  |  |  |  |
|  |  |  |  |  |  |  |  |  |  |
|  | XP_002612924.1 |  |  | P78563.1 |  | dsrm |  |  | dsrm |
|  |  |  |  |  |  | dsrm |  |  | dsrm |
|  |  |  |  |  |  |  |  |  | A_deamin |
|  |  |  |  |  |  |  |  |  |  |
|  |  |  |  |  |  |  |  |  |  |
| 329040_PRF0 | XP_002612907.1 | GETC01124369.1 | JT903429.1 | Q9UBK8.3 | Flavodoxin_1 | Flavodoxin_1 | Flavodoxin_1 |  | Flavodoxin_1 |
|  |  |  |  |  | FAD_binding_1 | FAD_binding_1 | FAD_binding_1 |  | FAD_binding_1 |
|  |  |  |  |  | NAD_binding_1 | NAD_binding_1 | NAD_binding_1 | NAD_binding_1 | NAD_binding_1 |
|  |  |  |  |  |  |  |  |  |  |
|  |  |  | JT869579.1 | Q9UBK8.3 |  |  |  | Flavodoxin_1 | Flavodoxin_1 |
|  |  |  |  |  |  |  |  |  | FAD_binding_1 |
|  |  |  |  |  |  |  |  |  | NAD_binding_1 |
|  |  |  |  |  |  |  |  |  |  |
| 329370_PRF0 | XP_002611195.1 | GETC01132691.1 | JT847778.1; JT867283.1;  JT852023.1 | P09917.2 |  | Catalase |  |  |  |
|  |  |  |  |  | PLAT | PLAT | PLAT |  | PLAT |
|  |  |  |  |  | Lipoxygenase | Lipoxygenase | Lipoxygenase |  | Lipoxygenase |
|  |  |  |  |  |  |  |  |  |  |
| 329970_PRF0 | XP_002606233.1 | GETC01136288.1 |  | P49619.3 | DAG_kinase_N | DAG_kinase_N | DAG_kinase_N |  | DAG_kinase_N |
|  |  |  |  |  | EF-hand_7 | EF-hand_7 | EF-hand_7 |  | EF-hand_7 |
|  |  |  |  |  | C1_1 | C1_1 | C1_1 |  | C1_1 |
|  |  |  |  |  | C1_1 | C1_1 | C1_1 |  | C1_1 |
|  |  |  |  |  | DAGK_cat | DAGK_cat | DAGK_cat |  | DAGK_cat |
|  |  |  |  |  | DAGK_acc | DAGK_acc | DAGK_acc |  | DAGK_acc |
|  |  |  |  |  |  |  |  |  |  |
| 330040_PRF0 |  | GESY01079088.1 | JT889425.1 | Q13873.2 | Activin_recp |  | Activin_recp |  | Activin_recp |
|  |  |  |  |  | Pkinase |  | Pkinase |  | Pkinase |
|  |  |  |  |  |  |  |  |  |  |
| 330210_PRF0 | XP_002602990.1 | GESY01078193.1 | JT889485.1 | O75746.2 | EF-hand | EF-hand | EF-hand | EF-hand | EF-hand |
|  |  |  |  |  | Mito_carr | Mito_carr | Mito_carr |  | Mito_carr |
|  |  |  |  |  | Mito_carr | Mito_carr | Mito_carr |  | Mito_carr |
|  |  |  |  |  | Mito_carr | Mito_carr | Mito_carr |  | Mito_carr |
|  |  |  |  |  |  | Peptidase M2 |  |  |  |
|  |  |  |  |  |  |  |  |  |  |
|  |  |  | JT900124.1 | O75746.2 |  |  |  |  | EF-hand |
|  |  |  |  |  |  |  |  | Mito_carr | Mito_carr |
|  |  |  |  |  |  |  |  | Mito_carr | Mito_carr |
|  |  |  |  |  |  |  |  |  | Mito_carr |
|  |  |  |  |  |  |  |  |  |  |
|  |  |  |  |  |  |  |  |  |  |
|  |  |  | JT896984.1; JT887996.1 | O75746.2 |  |  |  |  | EF-hand |
|  |  |  |  |  |  |  |  |  | Mito_carr |
|  |  |  |  |  |  |  |  |  | Mito_carr |
|  |  |  |  |  |  |  |  | Mito_carr | Mito_carr |
|  |  |  |  |  |  |  |  |  |  |
