# Supplementary material for: Morphological Stasis and Proteome Innovation in Cephalochordates
Source: Genes (Basel). 2018 Jul 16;9(7):353. doi: 10.3390/genes9070353 (PMC6071037; doi:10.3390/genes9070353)
Supplement: Supplementary file 1 [file genes-09-00353-s001.zip › genes-314079-supplementary-table S2.docx]

Supplementary Material: Morphological Stasis and Proteome Innovation
in Cephalochordates

László Bányai, Krisztina Kerekes, Mária Trexler and László Patthy

**Table S2.** Comparison of the domain architectures of key developmental proteins of lancelets with those of their human Swiss-Prot orthologs Key developmental proteins involved in determining anteroposterior, dorsoventral, and left–right assymetric development of *Branchiostoma. belcheri* were selected, and their lancelet and human Swiss-Prot orthologs were identified by the reciprocal best-hit method. Domain architectures, defined as the linear sequence of Pfam-A domains, were determined with Pfam. The first five columns of the table list the sequence IDs of predicted proteins (*B. belcheri and B. floridae*), sequence IDs of trancripts of orthologous protein-coding genes (*Asymmetron lucayanum* and *B. lanceolatum*), and of IDs of human Swiss-Prot entries. The last five columns of the table indicate the PfamA domains present in the various entries. Cells containing entries whose domain architecture is identical with those of orthologs in the Swiss-Prot database are highlighted in green.

| **Sequence ID** | | | | | **Domain architecture** | | | | |
| --- | --- | --- | --- | --- | --- | --- | --- | --- | --- |
| *B. belcheri* | *B. floridae* | *A. lucayanum* | *B. lanceolatum* | *H. sapiens* | *B. belcheri* | *B. floridae* | *A. lucayanum* | *B. lanceolatum* | *H. sapiens* |
|  |  |  |  |  |  |  |  |  |  |
| Bb_091450F | XP_002590390.1 | GETC01100775.1 | JT872400.1 | O15178.1 | T-box | T-box | T-box | T-box | T-box |
|  |  |  |  |  |  |  |  |  |  |
|  |  |  | JT860937.1; JT891285.1; JT874053.1 | O15178.1 |  |  |  |  | T-box |
|  |  |  |  |  |  |  |  |  |  |
| Bb_056190F | XP_002610790.1 | GETC01124733.1 | JT884202.1 | Q9H2X0.2 | VWC | VWC | VWC | VWC | VWC |
|  |  |  |  |  | CHRD | CHRD | CHRD |  | CHRD |
|  |  |  |  |  |  | CHRD | CHRD |  | CHRD |
|  |  |  |  |  |  | CHRD | CHRD |  | CHRD |
|  |  |  |  |  | CHRD | CHRD | CHRD |  | CHRD |
|  |  |  |  |  | VWC | VWC | VWC | VWC | VWC |
|  |  |  |  |  | VWC | VWC | VWC |  | VWC |
|  |  |  |  |  |  | VWC | VWC |  | VWC |
|  |  |  |  |  | PKD_channel |  |  |  |  |
|  |  |  |  |  |  |  |  |  |  |
|  |  |  | JT869941.1 | Q9H2X0.2 |  |  |  |  | VWC |
|  |  |  |  |  |  |  |  |  | CHRD |
|  |  |  |  |  |  |  |  |  | CHRD |
|  |  |  |  |  |  |  |  |  | CHRD |
|  |  |  |  |  |  |  |  |  | CHRD |
|  |  |  |  |  |  |  |  |  | VWC |
|  |  |  |  |  |  |  |  | VWC | VWC |
|  |  |  |  |  |  |  |  | VWC | VWC |
|  |  |  |  |  |  |  |  |  |  |
|  |  |  |  |  |  |  |  |  |  |
|  |  |  | JT860192.1 | Q9H2X0.2 |  |  |  |  | VWC |
|  |  |  |  |  |  |  |  |  | CHRD |
|  |  |  |  |  |  |  |  |  | CHRD |
|  |  |  |  |  |  |  |  |  | CHRD |
|  |  |  |  |  |  |  |  |  | CHRD |
|  |  |  |  |  |  |  |  | VWC | VWC |
|  |  |  |  |  |  |  |  |  | VWC |
|  |  |  |  |  |  |  |  |  | VWC |
|  |  |  |  |  |  |  |  |  |  |
| Bb_183350R | XP_002592105.1 | GETC01091836.1 | JT868007.1 | Q15465.1;  Q14623.4 | HH_signal | HH_signal | HH_signal | HH_signal | HH_signal |
|  |  |  |  |  | Hint | Hint | Hint | Hint | Hint |
|  |  |  |  |  |  |  |  |  |  |
|  |  |  | JT879422.1 | Q15465.1;  Q14623.4 |  |  |  |  | HH_signal |
|  |  |  |  |  |  |  |  | Hint | Hint |
|  |  |  |  |  |  |  |  |  |  |
| Bb_148630R | XP_002603845.1 | GETC01045908.1 | JT854662.1 | P18146.1 |  |  |  |  | DUF3446 |
|  |  |  |  |  | zf-C2H2 | zf-C2H2 | zf-C2H2 | zf-C2H2 | zf-C2H2 |
|  |  |  |  |  | zf-C2H2 | zf-C2H2 |  | zf-C2H2 | zf-C2H2 |
|  |  |  |  |  | zf-C2H2 | zf-C2H2 |  | zf-C2H2 | zf-C2H2 |
|  |  |  |  |  |  |  |  |  |  |
| Bb_115730R | XP_002603233.1 | GESY01026091.1 | JT899478.1 | P12829.3;  P60660.2;  P05976.3 | EF-hand_6 | EF-hand_6 | EF-hand_6 | EF-hand_6 | EF-hand_6 |
|  |  |  |  |  |  |  |  |  |  |
| Bb_010630F | XP_002604822.1 | GETC01121295.1 | JT884949.1 | P15172.3;  P13349.2 | Basic | Basic | Basic | Basic | Basic |
|  |  |  |  |  | HLH | HLH | HLH | HLH | HLH |
|  |  |  |  |  | Myf5 | Myf5 | Myf5 | Myf5 | Myf5 |
|  |  |  |  |  |  |  |  |  |  |
| Bb_134960R | XP_002596244.1 | GETC01061359.1 | JT856131.1 | Q92886.2;  Q9H2A3.2;  Q9Y4Z2.2 | HLH | HLH | HLH | HLH | HLH |
|  |  |  |  |  |  |  |  |  |  |
| Bb_002990R | XP_002610947.1 | GETC01064561.1 | JT881914.1 | Q96S42.2 | TGFb_propeptide | TGFb_propeptide | TGFb_propeptide | TGFb_propeptide | TGFb_propeptide |
|  |  |  |  |  | TGF_beta | TGF_beta | TGF_beta |  | TGF_beta |
|  |  |  |  |  |  |  |  |  |  |
|  |  |  | JT899582.1 |  |  |  |  |  | TGFb_propeptide |
|  |  |  |  |  |  |  |  | TGF_beta | TGF_beta |
|  |  |  |  |  |  |  |  |  |  |
| Bb_012650F |  | GETC01137287.1 | JT898607.1 | P46531.4 | EGF |  | EGF | EGF | EGF |
|  |  |  |  |  | EGF |  | EGF | EGF | EGF |
|  |  |  |  |  | EGF |  | EGF | EGF | EGF |
|  |  |  |  |  | EGF |  | EGF | EGF | EGF |
|  |  |  |  |  | EGF_CA |  | EGF_CA | EGF_CA | EGF_CA |
|  |  |  |  |  | EGF |  | EGF | EGF | EGF |
|  |  |  |  |  | EGF |  | EGF | EGF | EGF |
|  |  |  |  |  | EGF_CA |  | EGF_CA | EGF_CA | EGF_CA |
|  |  |  |  |  | EGF |  | EGF |  | EGF |
|  |  |  |  |  | EGF |  | EGF |  | EGF |
|  |  |  |  |  | EGF_CA |  | EGF_CA |  | EGF_CA |
|  |  |  |  |  | EGF |  | EGF |  | EGF |
|  |  |  |  |  | EGF |  | EGF |  | EGF |
|  |  |  |  |  | EGF |  |  |  |  |
|  |  |  |  |  | EGF |  |  |  |  |
|  |  |  |  |  | EGF |  |  |  |  |
|  |  |  |  |  | EGF |  |  |  |  |
|  |  |  |  |  | EGF_CA |  |  |  |  |
|  |  |  |  |  | EGF |  |  |  |  |
|  |  |  |  |  | EGF |  |  |  |  |
|  |  |  |  |  | EGF_CA |  |  |  |  |
|  |  |  |  |  | EGF |  |  |  |  |
|  |  |  |  |  | EGF |  | EGF |  | EGF |
|  |  |  |  |  | EGF |  | EGF |  | EGF |
|  |  |  |  |  | EGF |  | EGF |  | EGF |
|  |  |  |  |  | EGF |  | EGF |  | EGF |
|  |  |  |  |  | EGF |  | EGF |  | EGF |
|  |  |  |  |  | EGF |  | EGF |  | EGF |
|  |  |  |  |  | EGF |  | EGF |  | EGF |
|  |  |  |  |  | EGF |  | EGF |  | EGF |
|  |  |  |  |  | EGF |  | EGF |  | EGF |
|  |  |  |  |  | EGF |  |  |  | EGF_CA |
|  |  |  |  |  | EGF |  |  |  | EGF |
|  |  |  |  |  | EGF |  |  |  | EGF |
|  |  |  |  |  | EGF |  |  |  | EGF |
|  |  |  |  |  | EGF |  |  |  | EGF |
|  |  |  |  |  | EGF |  |  |  | EGF |
|  |  |  |  |  | EGF |  |  |  | EGF |
|  |  |  |  |  | EGF |  |  |  | EGF |
|  |  |  |  |  | EGF |  | EGF |  | EGF |
|  |  |  |  |  | EGF |  | EGF |  | EGF |
|  |  |  |  |  | EGF |  | EGF |  | EGF |
|  |  |  |  |  | EGF |  | EGF |  | EGF |
|  |  |  |  |  | EGF |  | EGF |  | EGF |
|  |  |  |  |  | EGF |  | EGF |  | EGF |
|  |  |  |  |  | Notch |  | Notch |  | Notch |
|  |  |  |  |  | Notch |  | Notch |  | Notch |
|  |  |  |  |  | Notch |  | Notch |  | Notch |
|  |  |  |  |  | NOD |  | NOD |  | NOD |
|  |  |  |  |  | NODP |  | NODP |  | NODP |
|  |  |  |  |  | Ank_2 |  | Ank_2 |  | Ank_2 |
|  |  |  |  |  | Ank_2 |  | Ank_2 |  | Ank_2 |
|  |  |  |  |  |  |  |  |  | DUF3454 |
|  |  |  |  |  |  |  |  |  |  |
|  |  | GESY01084869.1 |  | P46531.4 |  |  |  |  | EGF |
|  |  |  |  |  |  |  |  |  | EGF |
|  |  |  |  |  |  |  |  |  | EGF |
|  |  |  |  |  |  |  |  |  | EGF |
|  |  |  |  |  |  |  |  |  | EGF_CA |
|  |  |  |  |  |  |  |  |  | EGF |
|  |  |  |  |  |  |  |  |  | EGF |
|  |  |  |  |  |  |  |  |  | EGF_CA |
|  |  |  |  |  |  |  |  |  | EGF |
|  |  |  |  |  |  |  |  |  | EGF |
|  |  |  |  |  |  |  |  |  | EGF_CA |
|  |  |  |  |  |  |  |  |  | EGF |
|  |  |  |  |  |  |  |  |  | EGF |
|  |  |  |  |  |  |  |  |  | EGF |
|  |  |  |  |  |  |  |  |  | EGF |
|  |  |  |  |  |  |  |  |  | EGF |
|  |  |  |  |  |  |  |  |  | EGF |
|  |  |  |  |  |  |  |  |  | EGF |
|  |  |  |  |  |  |  |  |  | EGF |
|  |  |  |  |  |  |  |  |  | EGF |
|  |  |  |  |  |  |  |  |  | EGF |
|  |  |  |  |  |  |  |  |  | EGF |
|  |  |  |  |  |  |  | EGF |  | EGF_CA |
|  |  |  |  |  |  |  | EGF |  | EGF |
|  |  |  |  |  |  |  | EGF |  | EGF |
|  |  |  |  |  |  |  | EGF |  | EGF |
|  |  |  |  |  |  |  | EGF |  | EGF |
|  |  |  |  |  |  |  | EGF |  | EGF |
|  |  |  |  |  |  |  | EGF |  | EGF |
|  |  |  |  |  |  |  | EGF |  | EGF |
|  |  |  |  |  |  |  | EGF |  | EGF |
|  |  |  |  |  |  |  | EGF |  | EGF |
|  |  |  |  |  |  |  | EGF |  | EGF |
|  |  |  |  |  |  |  | EGF |  | EGF |
|  |  |  |  |  |  |  | EGF |  | EGF |
|  |  |  |  |  |  |  | EGF |  | EGF |
|  |  |  |  |  |  |  | Notch |  | Notch |
|  |  |  |  |  |  |  | Notch |  | Notch |
|  |  |  |  |  |  |  | Notch |  | Notch |
|  |  |  |  |  |  |  | NOD |  | NOD |
|  |  |  |  |  |  |  | NODP |  | NODP |
|  |  |  |  |  |  |  | Ank_2 |  | Ank_2 |
|  |  |  |  |  |  |  | Ank_2 |  | Ank_2 |
|  |  |  |  |  |  |  | DUF3454 |  | DUF3454 |
|  |  |  |  |  |  |  |  |  |  |
|  |  |  | JT857162.1 | P46531.4 |  |  |  |  | EGF |
|  |  |  |  |  |  |  |  |  | EGF |
|  |  |  |  |  |  |  |  |  | EGF |
|  |  |  |  |  |  |  |  |  | EGF |
|  |  |  |  |  |  |  |  |  | EGF_CA |
|  |  |  |  |  |  |  |  |  | EGF |
|  |  |  |  |  |  |  |  |  | EGF |
|  |  |  |  |  |  |  |  |  | EGF_CA |
|  |  |  |  |  |  |  |  |  | EGF |
|  |  |  |  |  |  |  |  |  | EGF |
|  |  |  |  |  |  |  |  |  | EGF_CA |
|  |  |  |  |  |  |  |  |  | EGF |
|  |  |  |  |  |  |  |  |  | EGF |
|  |  |  |  |  |  |  |  |  | EGF |
|  |  |  |  |  |  |  |  | EGF | EGF |
|  |  |  |  |  |  |  |  | EGF | EGF |
|  |  |  |  |  |  |  |  | EGF | EGF |
|  |  |  |  |  |  |  |  | EGF | EGF |
|  |  |  |  |  |  |  |  | EGF | EGF |
|  |  |  |  |  |  |  |  | EGF | EGF |
|  |  |  |  |  |  |  |  | EGF | EGF |
|  |  |  |  |  |  |  |  | EGF | EGF |
|  |  |  |  |  |  |  |  |  | EGF_CA |
|  |  |  |  |  |  |  |  |  | EGF |
|  |  |  |  |  |  |  |  |  | EGF |
|  |  |  |  |  |  |  |  |  | EGF |
|  |  |  |  |  |  |  |  |  | EGF |
|  |  |  |  |  |  |  |  |  | EGF |
|  |  |  |  |  |  |  |  |  | EGF |
|  |  |  |  |  |  |  |  |  | EGF |
|  |  |  |  |  |  |  |  |  | EGF |
|  |  |  |  |  |  |  |  |  | EGF |
|  |  |  |  |  |  |  |  |  | EGF |
|  |  |  |  |  |  |  |  |  | EGF |
|  |  |  |  |  |  |  |  |  | EGF |
|  |  |  |  |  |  |  |  |  | EGF |
|  |  |  |  |  |  |  |  |  | Notch |
|  |  |  |  |  |  |  |  |  | Notch |
|  |  |  |  |  |  |  |  |  | Notch |
|  |  |  |  |  |  |  |  |  | NOD |
|  |  |  |  |  |  |  |  |  | NODP |
|  |  |  |  |  |  |  |  |  | Ank_2 |
|  |  |  |  |  |  |  |  |  | Ank_2 |
|  |  |  |  |  |  |  |  |  | DUF3454 |
|  |  |  |  |  |  |  |  |  |  |
|  |  |  | JT855407.1 | P46531.4 |  |  |  |  | EGF |
|  |  |  |  |  |  |  |  |  | EGF |
|  |  |  |  |  |  |  |  |  | EGF |
|  |  |  |  |  |  |  |  |  | EGF |
|  |  |  |  |  |  |  |  |  | EGF_CA |
|  |  |  |  |  |  |  |  |  | EGF |
|  |  |  |  |  |  |  |  |  | EGF |
|  |  |  |  |  |  |  |  |  | EGF_CA |
|  |  |  |  |  |  |  |  |  | EGF |
|  |  |  |  |  |  |  |  |  | EGF |
|  |  |  |  |  |  |  |  |  | EGF_CA |
|  |  |  |  |  |  |  |  |  | EGF |
|  |  |  |  |  |  |  |  |  | EGF |
|  |  |  |  |  |  |  |  |  | EGF |
|  |  |  |  |  |  |  |  |  | EGF |
|  |  |  |  |  |  |  |  |  | EGF |
|  |  |  |  |  |  |  |  |  | EGF |
|  |  |  |  |  |  |  |  |  | EGF |
|  |  |  |  |  |  |  |  |  | EGF |
|  |  |  |  |  |  |  |  |  | EGF |
|  |  |  |  |  |  |  |  |  | EGF |
|  |  |  |  |  |  |  |  |  | EGF |
|  |  |  |  |  |  |  |  |  | EGF_CA |
|  |  |  |  |  |  |  |  | EGF | EGF |
|  |  |  |  |  |  |  |  | EGF | EGF |
|  |  |  |  |  |  |  |  | EGF | EGF |
|  |  |  |  |  |  |  |  | EGF | EGF |
|  |  |  |  |  |  |  |  | EGF | EGF |
|  |  |  |  |  |  |  |  |  | EGF |
|  |  |  |  |  |  |  |  |  | EGF |
|  |  |  |  |  |  |  |  |  | EGF |
|  |  |  |  |  |  |  |  |  | EGF |
|  |  |  |  |  |  |  |  |  | EGF |
|  |  |  |  |  |  |  |  |  | EGF |
|  |  |  |  |  |  |  |  |  | EGF |
|  |  |  |  |  |  |  |  |  | EGF |
|  |  |  |  |  |  |  |  |  | Notch |
|  |  |  |  |  |  |  |  |  | Notch |
|  |  |  |  |  |  |  |  |  | Notch |
|  |  |  |  |  |  |  |  |  | NOD |
|  |  |  |  |  |  |  |  |  | NODP |
|  |  |  |  |  |  |  |  |  | Ank_2 |
|  |  |  |  |  |  |  |  |  | Ank_2 |
|  |  |  |  |  |  |  |  |  | DUF3454 |
|  |  |  |  |  |  |  |  |  |  |
|  |  |  | JT889144.1 | P46531.4 |  |  |  |  | EGF |
|  |  |  |  |  |  |  |  |  | EGF |
|  |  |  |  |  |  |  |  |  | EGF |
|  |  |  |  |  |  |  |  |  | EGF |
|  |  |  |  |  |  |  |  |  | EGF_CA |
|  |  |  |  |  |  |  |  |  | EGF |
|  |  |  |  |  |  |  |  |  | EGF |
|  |  |  |  |  |  |  |  |  | EGF_CA |
|  |  |  |  |  |  |  |  |  | EGF |
|  |  |  |  |  |  |  |  |  | EGF |
|  |  |  |  |  |  |  |  |  | EGF_CA |
|  |  |  |  |  |  |  |  |  | EGF |
|  |  |  |  |  |  |  |  |  | EGF |
|  |  |  |  |  |  |  |  |  | EGF |
|  |  |  |  |  |  |  |  |  | EGF |
|  |  |  |  |  |  |  |  |  | EGF |
|  |  |  |  |  |  |  |  |  | EGF |
|  |  |  |  |  |  |  |  |  | EGF |
|  |  |  |  |  |  |  |  |  | EGF |
|  |  |  |  |  |  |  |  |  | EGF |
|  |  |  |  |  |  |  |  |  | EGF |
|  |  |  |  |  |  |  |  |  | EGF |
|  |  |  |  |  |  |  |  |  | EGF_CA |
|  |  |  |  |  |  |  |  |  | EGF |
|  |  |  |  |  |  |  |  |  | EGF |
|  |  |  |  |  |  |  |  |  | EGF |
|  |  |  |  |  |  |  |  |  | EGF |
|  |  |  |  |  |  |  |  |  | EGF |
|  |  |  |  |  |  |  |  |  | EGF |
|  |  |  |  |  |  |  |  |  | EGF |
|  |  |  |  |  |  |  |  |  | EGF |
|  |  |  |  |  |  |  |  |  | EGF |
|  |  |  |  |  |  |  |  |  | EGF |
|  |  |  |  |  |  |  |  |  | EGF |
|  |  |  |  |  |  |  |  |  | EGF |
|  |  |  |  |  |  |  |  | EGF | EGF |
|  |  |  |  |  |  |  |  | Notch | Notch |
|  |  |  |  |  |  |  |  | Notch | Notch |
|  |  |  |  |  |  |  |  | Notch | Notch |
|  |  |  |  |  |  |  |  | NOD | NOD |
|  |  |  |  |  |  |  |  |  | NODP |
|  |  |  |  |  |  |  |  |  | Ank_2 |
|  |  |  |  |  |  |  |  |  | Ank_2 |
|  |  |  |  |  |  |  |  |  | DUF3454 |
|  |  |  |  |  |  |  |  |  |  |
|  |  |  | JT855456.1 | P46531.4 |  |  |  |  | EGF |
|  |  |  |  |  |  |  |  |  | EGF |
|  |  |  |  |  |  |  |  |  | EGF |
|  |  |  |  |  |  |  |  |  | EGF |
|  |  |  |  |  |  |  |  |  | EGF_CA |
|  |  |  |  |  |  |  |  |  | EGF |
|  |  |  |  |  |  |  |  |  | EGF |
|  |  |  |  |  |  |  |  |  | EGF_CA |
|  |  |  |  |  |  |  |  |  | EGF |
|  |  |  |  |  |  |  |  |  | EGF |
|  |  |  |  |  |  |  |  |  | EGF_CA |
|  |  |  |  |  |  |  |  |  | EGF |
|  |  |  |  |  |  |  |  |  | EGF |
|  |  |  |  |  |  |  |  |  | EGF |
|  |  |  |  |  |  |  |  |  | EGF |
|  |  |  |  |  |  |  |  |  | EGF |
|  |  |  |  |  |  |  |  |  | EGF |
|  |  |  |  |  |  |  |  |  | EGF |
|  |  |  |  |  |  |  |  |  | EGF |
|  |  |  |  |  |  |  |  |  | EGF |
|  |  |  |  |  |  |  |  |  | EGF |
|  |  |  |  |  |  |  |  |  | EGF |
|  |  |  |  |  |  |  |  |  | EGF_CA |
|  |  |  |  |  |  |  |  |  | EGF |
|  |  |  |  |  |  |  |  |  | EGF |
|  |  |  |  |  |  |  |  |  | EGF |
|  |  |  |  |  |  |  |  |  | EGF |
|  |  |  |  |  |  |  |  |  | EGF |
|  |  |  |  |  |  |  |  |  | EGF |
|  |  |  |  |  |  |  |  |  | EGF |
|  |  |  |  |  |  |  |  |  | EGF |
|  |  |  |  |  |  |  |  |  | EGF |
|  |  |  |  |  |  |  |  |  | EGF |
|  |  |  |  |  |  |  |  |  | EGF |
|  |  |  |  |  |  |  |  |  | EGF |
|  |  |  |  |  |  |  |  |  | EGF |
|  |  |  |  |  |  |  |  |  | Notch |
|  |  |  |  |  |  |  |  |  | Notch |
|  |  |  |  |  |  |  |  |  | Notch |
|  |  |  |  |  |  |  |  |  | NOD |
|  |  |  |  |  |  |  |  |  | NODP |
|  |  |  |  |  |  |  |  |  | Ank_2 |
|  |  |  |  |  |  |  |  |  | Ank_2 |
|  |  |  |  |  |  |  |  | DUF3454 | DUF3454 |
|  |  |  |  |  |  |  |  |  |  |
|  | XP_002610672.1 | GETC01072070.1 | JT856097.1 | P32243.1;  P32242.1 |  | Homeobox | Homeobox | Homeobox | Homeobox |
|  |  |  |  |  |  | TF_Otx | TF_Otx | TF_Otx | TF_Otx |
|  |  |  |  |  |  |  |  |  |  |
| Bb_055950F | XP_002610806.1 | GETC01050032.1 | JT881722.1 | P23759.4;  P23760.2 | PAX | PAX | PAX | PAX | PAX |
|  |  |  |  |  | Homeobox | Homeobox | Homeobox |  | Homeobox |
|  |  |  |  |  |  |  |  |  | Pax7 |
|  |  |  |  |  | PAX |  |  |  |  |
|  |  |  |  |  | Homeobox |  |  |  |  |
|  |  |  |  |  |  |  |  |  |  |
|  |  |  | JT905548.1 | P23759.4;  P23760.2 |  |  |  |  | PAX |
|  |  |  |  |  |  |  |  | Homeobox | Homeobox |
|  |  |  |  |  |  |  |  |  | Pax7 |
|  |  |  |  |  |  |  |  |  |  |
|  |  |  | JT860590.1 | P23759.4;  P23760.2 |  |  |  |  | PAX |
|  |  |  |  |  |  |  |  |  | Homeobox |
|  |  |  |  |  |  |  |  |  | Pax7 |
|  |  |  |  |  |  |  |  |  |  |
|  |  | GETC01131929.1 |  | P23759.4;  P23760.2 |  |  |  |  | PAX |
|  |  |  |  |  |  |  | Homeobox |  | Homeobox |
|  |  |  |  |  |  |  |  |  | Pax7 |
|  |  |  |  |  |  |  |  |  |  |
| Bb_302430F |  | GETC01078212.1 | JT881722.1 | P55771.3;  P15863.4 | PAX |  | PAX | PAX | PAX |
|  |  |  |  |  |  |  |  |  |  |
| Bb_082490F | XP_002613740.1 | GETC01114416.1 | JT883756.1 | Q02962.4;  Q02548.1;  Q06710.2 | PAX | PAX | PAX |  | PAX |
|  |  |  |  |  | Homeobox | Homeobox | Homeobox | Homeobox | Homeobox |
|  |  |  |  |  | PAX2_C | PAX2_C | PAX2_C |  | PAX2_C |
|  |  |  |  |  |  |  |  |  |  |
|  |  |  |  |  |  |  |  |  |  |
| Bb_134850R | XP_002595953.1 | GETC01033312.1 | JT898531.1 | P78337.2;  Q99697.2;  O75364.1 |  | Homeobox | Homeobox | Homeobox | Homeobox |
|  |  |  |  |  |  | OAR | OAR | OAR | OAR |
|  |  |  |  |  |  |  |  |  |  |
|  |  |  | JT873335.1 | P78337.2;  Q99697.2;  O75364.1 |  |  |  |  |  |
|  |  |  |  |  |  |  |  |  |  |
| Bb_177200F | XP_002598513.1 | GETC01134136.1 | JT854638.1 | P56703.2 | wnt | wnt | wnt | wnt | wnt |
|  |  |  |  |  |  |  |  |  |  |
|  |  |  | JT891713.1 | P56703.2 |  |  |  |  | wnt |
|  |  |  |  |  |  |  |  |  |  |
| Bb_039350R | XP_002597279.1 | GESY01045804.1 | JT882280.1 | P41221.2;  Q9H1J7.2 | wnt | wnt | wnt | wnt | wnt |
|  |  |  |  |  |  |  |  |  |  |
| Bb_258720R | XP_002598625.1 | GETC01110690.1 | JT850115.1 | Q9Y6F9.2 | wnt | wnt | wnt | wnt | wnt |
|  |  |  |  |  |  |  |  |  |  |
| Bb_039420R | XP_002597288.1 | GETC01096614.1 |  | P56706.2;  O00755.2 | wnt | wnt | wnt |  | wnt |
|  |  |  |  |  |  |  |  |  |  |
| Bb_265390R |  | GETC01147402.1 | JT848368.1 | Q8N907.1;  O60565.1;  Q9H772.1 | DAN |  | DAN | DAN | DAN |
|  |  |  |  |  |  |  |  |  |  |
|  | XP_002589219.1 | GETC01098396.1 | JT881922.1 | O75610.1;  O00292.2 |  | TGFb_propeptide | TGFb_propeptide |  | TGFb_propeptide |
|  |  |  |  |  |  | TGF_beta | TGF_beta | TGF_beta | TGF_beta |
|  |  |  |  |  |  |  |  |  |  |
|  |  |  | JT884352.1 | O75610.1;  O00292.2 |  |  |  |  | TGFb_propeptide |
|  |  |  |  |  |  |  |  |  | TGF_beta |
|  |  |  |  |  |  |  |  |  |  |
| Bb_060350F | XP_002587160.1 | GESY01012154.1 | JT854265.1 | P28069.1;  P20264.2;  P20265.4;  P49335.2 | Pou | Pou | Pou | Pou | Pou |
|  |  |  |  |  | Homeobox | Homeobox | Homeobox | Homeobox | Homeobox |
|  |  |  |  |  | Profilin |  |  |  |  |
|  |  |  |  |  |  |  |  |  |  |
| Bb_250610F | XP_002591838.1 | GETC01044013.1 | JT854647.1 | Q9UBR4.2;  Q969G2.2;  Q9H2C1.1 | LIM | LIM | LIM | LIM | LIM |
|  |  |  |  |  | LIM | LIM | LIM | LIM | LIM |
|  |  |  |  |  | Homeobox | Homeobox | Homeobox | Homeobox | Homeobox |
|  |  |  |  |  |  |  |  |  |  |
| Bb_050390R | XP_002602111.1 | GETC01079967.1 | JT863995.1 | O94907.1;  Q9UBU2.1;  Q9UBT3.1 | Dickkopf_N | Dickkopf_N | Dickkopf_N | Prokineticin | Dickkopf_N |
|  |  |  |  |  |  |  |  |  |  |
| Bb_305780R | XP_002596858.1 | GETC01047898.1 | JT855141.1 | P12643.1;  P12644.1 | TGFb_propeptide | TGFb_propeptide | TGFb_propeptide | TGFb_propeptide | TGFb_propeptide |
|  |  |  |  |  | TGF_beta | TGF_beta | TGF_beta | TGF_beta | TGF_beta |
|  |  |  |  |  |  |  |  |  |  |
| Bb_033160F | XP_002588013.1 | GESY01031243.1 | JT854537.1 | P22003;  P22004;  P18075;  P34820 | TGFb_propeptide | TGFb_propeptide | TGFb_propeptide | TGFb_propeptide | TGFb_propeptide |
|  |  |  |  |  | TGF_beta | TGF_beta | TGF_beta | TGF_beta | TGF_beta |
|  |  |  |  |  |  |  |  |  |  |
|  | XP_002588012.1 |  |  | P22003;  P22004;  P18075;  P34820 |  | zf-C2H2 |  |  |  |
|  |  |  |  |  |  | zf-C2H2 |  |  |  |
|  |  |  |  |  |  | zf-C2H2 |  |  |  |
|  |  |  |  |  |  | zf-C2H2 |  |  |  |
|  |  |  |  |  |  | WD40 |  |  |  |
|  |  |  |  |  |  | WD40 |  |  |  |
|  |  |  |  |  |  | WD40 |  |  |  |
|  |  |  |  |  |  | WD40 |  |  |  |
|  |  |  |  |  |  | TGFb_propeptide |  |  | TGFb_propeptide |
|  |  |  |  |  |  |  |  |  | TGF_beta |
|  |  |  |  |  |  |  |  |  |  |
| Bb_147770R | XP_002608052.1 | GETC01070918.1 | JT855664.1 | Q15797.1;  Q99717.1 | MH1 |  | MH1 | MH1 | MH1 |
|  |  |  |  |  | MH2 |  | MH2 | MH2 | MH2 |
|  |  |  |  |  |  |  |  |  |  |
|  |  |  | JT883422.1 | Q15797.1;  Q99717.1 |  |  |  |  | MH1 |
|  |  |  |  |  |  |  |  | MH2 | MH2 |
|  |  |  |  |  |  |  |  |  |  |
|  |  |  | JT875987.1 | Q15797.1;  Q99717.1 |  |  |  |  | MH1 |
|  |  |  |  |  |  |  |  |  | MH2 |
|  |  |  |  |  |  |  |  |  |  |
| Bb_080180R | XP_002605517.1 | GESY01068021.1 | JT872924.1 | Q13485 | MH1 | MH1 | MH1 | MH1 | MH1 |
|  |  |  |  |  | MH2 | MH2 | MH2 |  | MH2 |
|  |  |  |  |  |  |  |  |  |  |
|  |  |  | JT886084.1 | Q13485 |  |  |  |  | MH1 |
|  |  |  |  |  |  |  |  |  | MH2 |
|  |  |  |  |  |  |  |  |  |  |
| Bb_238200F | XP_002602547.1 | GETC01071404.1 | JT854841.1 | O00358.3 | Forkhead | Forkhead | Forkhead | Forkhead | Forkhead |
|  |  |  |  |  |  |  |  |  |  |
|  |  |  | JT873731.1 | O00358.3 |  |  |  |  | Forkhead |
|  |  |  |  |  |  |  |  |  |  |
|  |  |  | JT857292.1 | O00358.3 |  |  |  |  | Forkhead |
|  |  |  |  |  |  |  |  |  |  |
|  |  |  | JT855481.1 | O00358.3 |  |  |  |  | Forkhead |
|  |  |  |  |  |  |  |  |  |  |
|  |  | GETC01127652.1 |  | O00358.3 |  |  |  |  | Forkhead |
|  |  |  |  |  |  |  |  |  |  |
| Bb_062520R | XP_002594291.1 | GESY01013097.1 | JT882905.1 | Q9C009.2 | Forkhead | Forkhead | Forkhead | Forkhead | Forkhead |
|  |  |  |  |  |  |  |  |  |  |
|  |  | GETC01056388.1 |  | Q9C009.2 |  |  | Forkhead |  | Forkhead |
|  |  |  |  |  |  |  |  |  |  |
|  |  | GESY01000330.1 |  | Q9C009.2 |  |  |  |  | Forkhead |
|  |  |  |  |  |  |  |  |  |  |
|  |  |  | JT894417.1 |  |  |  |  |  |  |
|  |  |  |  |  |  |  |  |  |  |
| Bb_108560F | XP_002589194.1 | GETC01073085.1 | JT884317.1 | P43699.1;  Q9H2Z4.3 | Homeobox | Homeobox | Homeobox | Homeobox | Homeobox |
|  |  |  |  |  |  |  |  |  |  |
|  |  | GETC01073083.1 |  | P43699.1;  Q9H2Z4.3 |  |  |  |  | Homeobox; |
|  |  |  |  |  |  |  |  |  |  |
|  |  |  | JT898481.1 | P43699.1;  Q9H2Z4.3 |  |  |  |  | Homeobox; |
|  |  |  |  |  |  |  |  |  |  |
| Bb_306640F | XP_002598626.1 | GETC01120369.1 | JT884311.1 | P04628 | wnt | wnt | wnt | wnt | wnt |
|  |  |  |  |  |  |  |  |  |  |
|  |  |  |  |  |  |  |  |  |  |
| Bb_172970F | XP_002603694.1 | GETC01025000.1 | JT850936.1 | P55075.1;  O60258.1;  O76093.1 | FGF | FGF | FGF | FGF | FGF |
|  |  |  |  |  |  |  |  |  |  |
|  |  |  | JT877008.1 | P55075.1;  O60258.1;  O76093.1 |  |  |  |  | FGF |
|  |  |  |  |  |  |  |  |  |  |
| Bb_082490F | XP_002613740.1 | GETC01114413.1 | JT883756.1 | Q02962;  Q02548;  Q06710 | PAX | PAX | PAX |  | PAX |
|  |  |  |  |  | Homeobox | Homeobox | Homeobox | Homeobox | Homeobox |
|  |  |  |  |  | Pax2_C | Pax2_C | Pax2_C |  | Pax2_C |
|  |  |  |  |  |  |  |  |  |  |
|  |  |  | JT856081.1 | Q02962;  Q02548;  Q06710 |  |  |  |  | PAX |
|  |  |  |  |  |  |  |  |  | Homeobox |
|  |  |  |  |  |  |  |  |  | Pax2_C |
|  |  |  |  |  |  |  |  |  |  |
| Bb_231480R | XP_002612940.1 | GETC01055780.1 | JT859615.1 | P19622.3;  Q05925.3 | Homeobox | Homeobox | Homeobox | Homeobox | Homeobox |
|  |  |  |  |  | Engrail_1_C_sig | Engrail_1_C_sig | Engrail_1_C_sig | Engrail_1_C_sig | Engrail_1_C_sig |
|  |  |  |  |  |  |  |  |  |  |
|  | XP_002610672.1 | GETC01072070.1 | JT856097.1 | P32243.1;  P32242.1;  O43186.1 |  | Homeobox | Homeobox | Homeobox | Homeobox |
|  |  |  |  |  |  | TF_Otx | TF_Otx | TF_Otx | TF_Otx |
|  |  |  |  |  |  |  |  |  |  |
| Bb_331680R=Bb_175690F | XP_002612719.1 | GETC01088425.1 | JT882234.1 | P49639.2;  P14653.2 | Homeobox | Homeobox | Homeobox | Homeobox | Homeobox |
|  |  |  |  |  |  |  |  |  |  |
| Bb_042330F | XP_002593745.1 | GETC01127396.1 | JT888081.1 | P40424;  P40425;  P40426 | PBC | PBC | PBC | PBC | PBC |
|  |  |  |  |  |  | PBC |  |  |  |
|  |  |  |  |  | Homeobox | Homeobox | Homeobox |  | Homeobox |
|  |  |  |  |  |  |  |  |  |  |
|  |  |  | JT894930.1 | P40424;  P40425;  P40426 |  |  |  |  | PBC |
|  |  |  |  |  |  |  |  | Homeobox | Homeobox |
|  |  |  |  |  |  |  |  |  |  |
