# Supplementary material for: Morphological Stasis and Proteome Innovation in Cephalochordates
Source: Genes (Basel). 2018 Jul 16;9(7):353. doi: 10.3390/genes9070353 (PMC6071037; doi:10.3390/genes9070353)
Supplement: Supplementary file 1 [file genes-09-00353-s001.zip › genes-314079-supplementary-table S3.docx]

Supplementary Material: Morphological Stasis and Proteome Innovation
in Cephalochordates

László Bányai, Krisztina Kerekes, Mária Trexler and László Patthy

**Table S3.** Comparison of the domain architectures of randomly selected lancelet proteins with novel domain architectures. Proteins of *Branchiostoma belcheri*, containing at least two Pfam-A domains, were randomly selected, and their lancelet orthologs were identified by the reciprocal best-hit method. The table compares the domain architectures of proteins that have no equivalents in the high quality Swiss-Prot database. Domain architectures, defined as the linear sequence of Pfam-A domains, were determined with Pfam. The first four columns of the table list the sequence IDs of pedicted proteins (*B. belcheri* and *B. floridae*), and sequence IDs of trancripts of orthologous protein-coding genes (*Asymmetron lucayanum* and *B. lanceolatum*). The last five columns of the table indicate the PfamA domains present in the various entries. Cells containing proteins whose structure does not violate any of the MisPred rules are highlighted in green.

| **Sequence ID** | | | | **Domain architecture** | | | |
| --- | --- | --- | --- | --- | --- | --- | --- |
| ***B. belcheri*** | ***B. floridae*** | ***A. lucayanum*** | ***B. lanceolatum*** | ***B. belcheri*** | ***B. floridae*** | ***A. lucayanum*** | ***B. lanceolatum*** |
|  |  |  |  |  |  |  |  |
| 000200_PFF0 | AAN62850.1 | GESY01041941.1 |  | V-set | V-set | V-set |  |
|  |  |  |  | CBM_14 | CBM_14 | CBM_14 |  |
|  |  |  |  |  |  |  |  |
| 001280_PFF0 | XP_002608265.1 | GESY01048909.1 |  | CBM_14 | CBM_14 | CBM_14 |  |
|  |  |  |  | KASH_CCD | KASH_CCD | KASH_CCD |  |
|  |  |  |  | ILEI | ILEI | ILEI |  |
|  |  |  |  |  |  |  |  |
| 017590_PFF0 | XP_002598432.1 | GETC01035413.1 |  | Cu-oxidase_3 | Cu-oxidase_3 |  |  |
|  |  |  |  | Cu-oxidase | Cu-oxidase | Cu-oxidase |  |
|  |  |  |  | Cu-oxidase_2 | Cu-oxidase_2 | Cu-oxidase_2 |  |
|  |  |  |  |  |  |  |  |
| 017700_PFF0 | XP_002598460.1 | GETC01136258.1 | JT893662.1 | Gal_Lectin | Gal_Lectin | Gal_Lectin |  |
|  |  |  |  | Gal_Lectin | Gal_Lectin | Gal_Lectin |  |
|  |  |  |  | GPS | GPS | GPS |  |
|  |  |  |  | 7tm_2 | 7tm_2 | 7tm_2 | 7tm_2 |
|  |  |  |  |  |  |  |  |
| 018300_PFF0 | XP_002588697.1 | GESY01072040.1 | JT854458.1 | Lectin_C | Lectin_C | Lectin_C | Lectin_C |
|  |  |  |  | fn2 | fn2 |  |  |
|  |  |  |  | fn2 | fn2 |  |  |
|  |  |  |  | fn2 | fn2 |  |  |
|  |  |  |  | fn2 | fn2 |  |  |
|  |  |  |  | fn2 | fn2 |  |  |
|  |  |  |  |  |  |  |  |
| 040970_PFF0 | XP_002596007.1 | GESY01068797.1 |  | kringle | kringle | kringle |  |
|  |  |  |  | kringle | kringle | kringle |  |
|  |  |  |  | trypsin | trypsin | trypsin |  |
|  |  |  |  |  |  |  |  |
| 068220_PFF0 | XP_002586877.1 | GESY01052491.1 | JT904011.1 | fn2 | fn2 | fn2 |  |
|  |  |  |  | fn2 | fn2 | fn2 |  |
|  |  |  |  | fn2 | fn2 | fn2 |  |
|  |  |  |  | fn2 | fn2 | fn2 |  |
|  |  |  |  | CUB | CUB | CUB |  |
|  |  |  |  | CUB | CUB | CUB | CUB |
|  |  |  |  |  |  |  |  |
| 080050_PFF0 | XP_002606926.1 | GETC01035867.1 | JT896252.1 | N-glycanase_N | N-glycanase_N | N-glycanase_N |  |
|  |  |  |  | N-glycanase_C | N-glycanase_C | N-glycanase_C |  |
|  |  |  |  |  |  |  |  |
| 124390_PFF0 |  | GETC01028934.1 | JT878227.1 | cEGF |  |  |  |
|  |  |  |  | FXa_inhibition |  |  |  |
|  |  |  |  | EGF_CA |  | EGF_CA |  |
|  |  |  |  | EGF_CA |  | EGF_CA |  |
|  |  |  |  | NIDO |  |  |  |
|  |  |  |  | VWD |  |  |  |
|  |  |  |  | EGF_CA |  |  |  |
|  |  |  |  | EGF_CA |  |  |  |
|  |  |  |  | cEGF |  |  |  |
|  |  |  |  | SEA |  |  |  |
|  |  |  |  | EGF_CA |  |  | EGF_CA |
|  |  |  |  |  |  |  |  |
| 124390_PFF0 |  | GETC01031440.1 |  | cEGF |  |  |  |
|  |  |  |  | FXa_inhibition |  |  |  |
|  |  |  |  | EGF_CA |  |  |  |
|  |  |  |  | EGF_CA |  |  |  |
|  |  |  |  | NIDO |  |  |  |
|  |  |  |  | VWD |  | VWD |  |
|  |  |  |  | EGF_CA |  |  |  |
|  |  |  |  | EGF_CA |  |  |  |
|  |  |  |  | cEGF |  |  |  |
|  |  |  |  | SEA |  |  |  |
|  |  |  |  | EGF_CA |  |  |  |
|  |  |  |  |  |  |  |  |
| 124390_PFF0 |  | GETC01009491.1 |  | cEGF |  |  |  |
|  |  |  |  | FXa_inhibition |  |  |  |
|  |  |  |  | EGF_CA |  |  |  |
|  |  |  |  | EGF_CA |  |  |  |
|  |  |  |  | NIDO |  |  |  |
|  |  |  |  | VWD |  |  |  |
|  |  |  |  | EGF_CA |  | EGF_CA |  |
|  |  |  |  | EGF_CA |  | EGF_CA |  |
|  |  |  |  | cEGF |  |  |  |
|  |  |  |  | SEA |  |  |  |
|  |  |  |  | EGF_CA |  |  |  |
|  |  |  |  |  |  |  |  |
| 124390_PFF0 |  | GETC01028688.1 |  | cEGF |  |  |  |
|  |  |  |  | FXa_inhibition |  |  |  |
|  |  |  |  | EGF_CA |  |  |  |
|  |  |  |  | EGF_CA |  |  |  |
|  |  |  |  | NIDO |  |  |  |
|  |  |  |  | VWD |  |  |  |
|  |  |  |  | EGF_CA |  |  |  |
|  |  |  |  | EGF_CA |  |  |  |
|  |  |  |  | cEGF |  |  |  |
|  |  |  |  | SEA |  |  |  |
|  |  |  |  | EGF_CA |  | EGF_CA |  |
|  |  |  |  |  |  |  |  |
| 157720_PFF0 | XP_002612291.1 | GETC01137028.1 | JT898889.1; JT895992.1;  JT897736.1; JT866935.1 |  | Troponin |  |  |
|  |  |  |  | HYR | HYR | HYR |  |
|  |  |  |  | Ephrin_rec_like | Ephrin_rec_like | Ephrin_rec_like |  |
|  |  |  |  |  |  |  |  |
| 159900_PFF0 | XP_002600642.1 | GETC01121910.1 |  | AAA_8 | AAA_8 | AAA_8 |  |
|  |  |  |  | MT | MT | MT |  |
|  |  |  |  | AAA_9 | AAA_9 | AAA_9 |  |
|  |  |  |  | Dynein_heavy | Dynein_heavy | Dynein_heavy |  |
|  |  |  |  |  |  |  |  |
| 167280_PRF0 | XP_002611740.1 |  |  | Lectin_C | Lectin_C |  |  |
|  |  |  |  | Kringle | Kringle |  |  |
|  |  |  |  | Somatomedin_B | Somatomedin_B |  |  |
|  |  |  |  | 7tm_2 | 7tm_2 |  |  |
|  |  |  |  |  |  |  |  |
| 173360_PRF0 | XP_002593467.1 | GESY01047447.1 | JT897220.1 | Astacin | Astacin | Astacin | Astacin |
|  |  |  |  | Kringle | Kringle | Kringle |  |
|  |  |  |  |  |  |  |  |
| 180420_PRF0 | XP_002609978.1 | GETC01072780.1 | JT858326.1 |  | 5-FTHF_cyc-lig | 5-FTHF_cyc-lig | 5-FTHF_cyc-lig |
|  |  |  |  | Somatomedin_B | Somatomedin_B |  |  |
|  |  |  |  |  | Somatomedin_B |  |  |
|  |  |  |  | 7tm_2 | 7tm_2 |  |  |
|  |  |  |  |  |  |  |  |
| 184730_PRF0 | XP_002596567.1 | GETC01152535.1 |  | Ldl_recept_a |  |  |  |
|  |  |  |  | Kringle |  |  |  |
|  |  |  |  | Kringle | Kringle | Kringle |  |
|  |  |  |  | 7tm_2 |  |  |  |
|  |  |  |  |  |  |  |  |
| 188610_PRF0 | XP_002609579.1 | GESY01021940.1 |  | ASC | ASC |  |  |
|  |  |  |  | F5_F8_type_C | F5_F8_type_C |  |  |
|  |  |  |  | F5_F8_type_C | F5_F8_type_C |  |  |
|  |  |  |  | Kringle | Kringle |  |  |
|  |  |  |  | ASC | ASC | ASC |  |
|  |  |  |  |  |  |  |  |
| 188610_PRF0 | XP_002609579.1 | GESY01001278.1 |  | ASC | ASC |  |  |
|  |  |  |  | F5_F8_type_C | F5_F8_type_C |  |  |
|  |  |  |  | F5_F8_type_C | F5_F8_type_C |  |  |
|  |  |  |  | Kringle | Kringle | Kringle |  |
|  |  |  |  | ASC | ASC | ASC |  |
|  |  |  |  |  |  |  |  |
| 188610_PRF0 | XP_002609579.1 | GETC01149215.1 |  | ASC | ASC |  |  |
|  |  |  |  | F5_F8_type_C | F5_F8_type_C |  |  |
|  |  |  |  | F5_F8_type_C | F5_F8_type_C |  |  |
|  |  |  |  | Kringle | Kringle | Kringle |  |
|  |  |  |  | ASC | ASC |  |  |
|  |  |  |  |  |  |  |  |
| 196740_PRF0 | XP_002601343.1 | GESY01088885.1 | JT865136.1 | Fibrinogen_C |  | Fibrinogen_C |  |
|  |  |  |  | Fibrinogen_C | Fibrinogen_C | Fibrinogen_C |  |
|  |  |  |  | I-set | I-set | I-set |  |
|  |  |  |  | fn3 | fn3 | fn3 |  |
|  |  |  |  | fn3 | fn3 | fn3 |  |
|  |  |  |  | fn3 | fn3 | fn3 |  |
|  |  |  |  | fn3 | fn3 | fn3 |  |
|  |  |  |  | Y_phosphatase |  | Y_phosphatase |  |
|  |  |  |  | Y_phosphatase |  | Y_phosphatase | Y_phosphatase |
|  |  |  |  |  |  |  |  |
| 196740_PRF0 | XP_002601342.1 |  |  | Fibrinogen_C |  |  |  |
|  |  |  |  | Fibrinogen_C |  |  |  |
|  |  |  |  | I-set |  |  |  |
|  |  |  |  | fn3 |  |  |  |
|  |  |  |  | fn3 |  |  |  |
|  |  |  |  | fn3 |  |  |  |
|  |  |  |  | fn3 |  |  |  |
|  |  |  |  | Y_phosphatase | Y_phosphatase |  |  |
|  |  |  |  | Y_phosphatase | Y_phosphatase |  |  |
|  |  |  |  |  |  |  |  |
| 196740_PRF0 | XP_002601344.1 |  |  | Fibrinogen_C | Fibrinogen_C |  |  |
|  |  |  |  | Fibrinogen_C |  |  |  |
|  |  |  |  | I-set |  |  |  |
|  |  |  |  | fn3 |  |  |  |
|  |  |  |  | fn3 |  |  |  |
|  |  |  |  | fn3 |  |  |  |
|  |  |  |  | fn3 |  |  |  |
|  |  |  |  | Y_phosphatase |  |  |  |
|  |  |  |  | Y_phosphatase |  |  |  |
|  |  |  |  |  |  |  |  |
| 200810_PRF0 | XP_002608508.1 | GESY01050654.1 |  | Astacin | Astacin | Astacin |  |
|  |  |  |  | CUB | CUB | CUB |  |
|  |  |  |  | MAM | MAM | MAM |  |
|  |  |  |  |  |  |  |  |
| 258300_PRF0 | XP_002605710.1 | GESY01085563.1 |  |  |  | Ig_3 |  |
|  |  |  |  |  |  | I-set |  |
|  |  |  |  |  |  | I-set |  |
|  |  |  |  |  |  | I-set |  |
|  |  |  |  |  |  | SRCR |  |
|  |  |  |  |  |  | Kringle |  |
|  |  |  |  |  |  | Kringle |  |
|  |  |  |  |  |  | Ldl_recept_a |  |
|  |  |  |  |  |  | PAN_1 |  |
|  |  |  |  | Ldl_recept_a |  | Ldl_recept_a |  |
|  |  |  |  | SRCR |  | SRCR |  |
|  |  |  |  | Ldl_recept_a |  | Ldl_recept_a |  |
|  |  |  |  | Trypsin | Trypsin | Trypsin |  |
|  |  |  |  |  |  |  |  |
| 258300_PRF0 | XP_002605711.1 |  |  | Ldl_recept_a |  |  |  |
|  |  |  |  | SRCR | SRCR |  |  |
|  |  |  |  | Ldl_recept_a |  |  |  |
|  |  |  |  | Trypsin |  |  |  |
|  |  |  |  |  |  |  |  |
| 258300_PRF0 |  | GETC01045113.1 |  | Ldl_recept_a |  | Ldl_recept_a |  |
|  |  |  |  | SRCR |  | SRCR |  |
|  |  |  |  | Ldl_recept_a |  |  |  |
|  |  |  |  | Trypsin |  |  |  |
|  |  |  |  |  |  |  |  |
| 258300_PRF0 |  | GETC01045114.1 |  | Ldl_recept_a |  |  |  |
|  |  |  |  | SRCR |  |  |  |
|  |  |  |  | Ldl_recept_a |  | Ldl_recept_a |  |
|  |  |  |  | Trypsin |  | Trypsin |  |
|  |  |  |  |  |  |  |  |
| 260260_PRF0 |  |  |  | BACK |  |  |  |
|  |  |  |  | Kelch_1 |  |  |  |
|  |  |  |  |  |  |  |  |
| 269420_PRF0 | XP_002605854.1 | GETC01135682.1 | JT850665.1 | CUB | CUB | CUB | CUB |
|  |  |  |  | Ldl_recept_a | Ldl_recept_a | Ldl_recept_a | Ldl_recept_a |
|  |  |  |  | Fz | Fz | Fz | Fz |
|  |  |  |  | Kringle | Kringle | Kringle |  |
|  |  |  |  |  | Sushi | Sushi |  |
|  |  |  |  |  |  | Neur_chan_LBD |  |
|  |  |  |  | Neur_chan_memb | Neur_chan_memb | Neur_chan_memb |  |
|  |  |  |  |  |  |  |  |
| 269420_PRF0 |  |  | JT855014.1 | CUB |  |  |  |
|  |  |  |  | Ldl_recept_a |  |  |  |
|  |  |  |  | Fz |  |  |  |
|  |  |  |  | Kringle |  |  |  |
|  |  |  |  |  |  |  | Sushi |
|  |  |  |  |  |  |  | Neur_chan_LBD |
|  |  |  |  | Neur_chan_memb |  |  |  |
|  |  |  |  |  |  |  |  |
| 283240_PRF0 | CAA06854.1 | GETC01135097.1 | JT870217.1 | Reeler | Reeler | Reeler | Reeler |
|  |  |  |  | Spond_N | Spond_N | Spond_N |  |
|  |  |  |  | fn3 | fn3 | fn3 |  |
|  |  |  |  | TSP_1 | TSP_1 | TSP_1 |  |
|  |  |  |  | fn3 | fn3 | fn3 |  |
|  |  |  |  | TSP_1 | TSP_1 | TSP_1 |  |
|  |  |  |  | TSP_1 | TSP_1 | TSP_1 |  |
|  |  |  |  |  |  |  |  |
| 283240_PRF0 |  |  | JT883615.1 | Reeler |  |  |  |
|  |  |  |  | Spond_N |  |  | Spond_N |
|  |  |  |  | fn3 |  |  | fn3 |
|  |  |  |  | TSP_1 |  |  | TSP_1 |
|  |  |  |  | fn3 |  |  |  |
|  |  |  |  | TSP_1 |  |  |  |
|  |  |  |  | TSP_1 |  |  |  |
|  |  |  |  |  |  |  |  |
| 283240_PRF0 |  |  | JT882352.1 | Reeler |  |  |  |
|  |  |  |  | Spond_N |  |  |  |
|  |  |  |  | fn3 |  |  |  |
|  |  |  |  | TSP_1 |  |  |  |
|  |  |  |  | fn3 |  |  | fn3 |
|  |  |  |  | TSP_1 |  |  | TSP_1 |
|  |  |  |  | TSP_1 |  |  | TSP_1 |
|  |  |  |  |  |  |  |  |
| 304470_PRF0 | XP_002587583.1 | GESY01066659.1 | JT857345.1 | Ldl_recept_a | Ldl_recept_a | Ldl_recept_a | Ldl_recept_a |
|  |  |  |  | VWA | VWA | VWA | VWA |
|  |  |  |  | Zona_pellucida | Zona_pellucida | Zona_pellucida |  |
|  |  |  |  |  |  |  |  |
| 304470_PRF0 |  |  | JT858265.1 | Ldl_recept_a |  |  |  |
|  |  |  |  | VWA |  |  | VWA |
|  |  |  |  | Zona_pellucida |  |  | Zona_pellucida |
|  |  |  |  |  |  |  |  |
| 320550_PRF0 | XP_002593208.1 | GESY01082967.1 |  | Kringle |  | Kringle |  |
|  |  |  |  | Kringle |  | Kringle |  |
|  |  |  |  | Lectin_C |  | Lectin_C |  |
|  |  |  |  | CUB |  | CUB |  |
|  |  |  |  | Lectin_C |  | Lectin_C |  |
|  |  |  |  | MAM |  | MAM |  |
|  |  |  |  | Lectin_C |  | Lectin_C |  |
|  |  |  |  | Kringle |  | Kringle |  |
|  |  |  |  | SRCR |  | SRCR |  |
|  |  |  |  |  |  |  |  |
| 329690_PRF0 | XP_002611153.1 | GESY01074984.1 |  | BTB | BTB | BTB |  |
|  |  |  |  | BTB | BTB | BTB |  |
|  |  |  |  | BACK | BACK | BACK |  |
|  |  |  |  | Kelch_1 | Kelch_1 | Kelch_1 |  |
|  |  |  |  | Kelch_1 | Kelch_1 | Kelch_1 |  |
|  |  |  |  | Kelch_1 | Kelch_1 | Kelch_1 |  |
|  |  |  |  | Kelch_1 | Kelch_1 | Kelch_1 |  |
|  |  |  |  |  |  |  |  |
| 329890_PRF0 | XP_002606023.1 | GETC01120975.1 |  | COR | COR |  |  |
|  |  |  |  | Death | Death | Death |  |
